# AAPM Medical physics practice guideline 15.A: Peer review in clinical physics

**DOI:** 10.1002/acm2.14151

**Published:** 2023-09-14

**Authors:** Per H. Halvorsen, Alan H. Baydush, Courtney R. Buckey, Navneeth Hariharan, Mary A. Keenan, Jeffrey P. Limmer, Kate E. Lofton, Robin A. Miller, Jeffrey M. Moirano, Joseph Och, Douglas E. Pfeiffer

**Affiliations:** ^1^ Varian Advanced Oncology Solutions Newton Massachusetts USA; ^2^ Novant Health Cancer Institute: Forsyth Medical Center Winston‐Salem North Carolina USA; ^3^ Mayo Clinic Arizona Phoenix Arizona USA; ^4^ Beth Israel ‐ Lahey Health Winchester Massachusetts USA; ^5^ Vanderbilt University Medical Center Nashville Tennessee USA; ^6^ Texas Oncology The Woodlands Texas USA; ^7^ Colorado Associates in Medical Physics Colorado Springs Colorado USA; ^8^ Northwest Medical Physics Center Lynnwood Washington USA; ^9^ University of Washington Seattle Washington USA; ^10^ Geisinger Medical Center Danville Pennsylvania USA; ^11^ Boulder Community Health Boulder Colorado USA

**Keywords:** MPPG, peer review, practice improvement

## Abstract

The American Association of Physicists in Medicine (AAPM) is a nonprofit professional society whose primary purposes are to advance the science, education, and professional practice of medical physics. The AAPM has more than 8000 members and is the principal organization of medical physicists in the United States.

The AAPM will periodically define new practice guidelines for medical physics practice to help advance the science of medical physics and to improve the quality of service to patients throughout the United States. Existing medical physics practice guidelines will be reviewed for the purpose of revision or renewal, as appropriate, on their fifth anniversary or sooner.

Each medical physics practice guideline represents a policy statement by the AAPM, has undergone a thorough consensus process in which it has been subjected to extensive review, and requires the approval of the Professional Council. The medical physics practice guidelines recognize that the safe and effective use of diagnostic and therapeutic radiology requires specific training, skills, and techniques, as described in each document. Reproduction or modification of the published practice guidelines and technical standards by those entities not providing these services is not authorized.

The following terms are used in the AAPM practice guidelines:

- Must and Must Not: Used to indicate that adherence to the recommendation is considered necessary to conform to this practice guideline. While must is the term to be used in the guidelines, if an entity that adopts the guideline has shall as the preferred term, the AAPM considers that must and shall have the same meaning.
- Should and Should Not: Used to indicate a prudent practice to which exceptions may occasionally be made in appropriate circumstances.

## 1 INTRODUCTION

### 1.1 Motivation

#### 1.1.1 Practice settings

Many medical physicists work as the only physicist in their facility (approximately 17% according to the 2020 AAPM Professional Survey Report^1^). This includes individuals in all aspects of medical physics. In such practice settings, the medical physicist does not have the continuous feedback of colleagues to refine and improve their skills and performance. Working alone, it is easy to become unaware of deficiencies in one's own work product. While continuing education (CE) is essential for professional development, even with adequate CE it is difficult for one person to keep track of all salient developments in their professional world.

Group practices can be subject to similar issues. While physicists in a group practice setting have the benefit of regular access to other physicists, groups can also become isolated from the broader medical physics society. They, too, can fail to evolve as standards of practice change. Additionally, individuals within the group might only have opportunities for growth in specific areas, or the group dynamic may not be conducive to constructive peer input.

Peer review (ideally from an external reviewer) can help to mitigate the situations described above. It is an important method for verifying patient safety and quality of care, with origins from the early days of Arab medicine more than a millennium ago.^2^, ^3^ It is an accepted system for ensuring professionalism and trust by providing an objective evaluation of a practitioner or clinical scientist's performance and professional practice by a qualified colleague.^4^, ^5^ As stated in the AAPM Task Group 103 report,^4^ “The purpose of the peer review process…is to enable a collegial exchange of professional ideas and promote a productive critique of the incumbent's clinical physics program with the aim of enhancing the program while ensuring conformance with regulations, professional guidelines, and established practice patterns.” As such, peer review can be beneficial to all clinical medical physicists.^6^ Note that this Practice Guideline focuses on professional peer review (evaluation of a colleague's professional practice), not “clinical peer review” or “medical peer review” (evaluation of medical decision making for specific patients).

#### 1.1.2 Behavioral aspect

Studies of human behavior^7^ generally agree that humans process information differently depending on the situation, and this can be broadly categorized into a performance mode and a learning mode. We spend most of our working hours in the performance mode, completing tasks efficiently but investing little focus on evaluating **how** the work is performed. The learning mode allows us to disengage from the daily routine and evaluate the broader context of our practice setting in order to identify opportunities for improvement. Professional peer review offers a mutual opportunity for a constructive learning mode with relevant feedback from a peer benefiting both the incumbent physicist and the reviewer.

The American Medical Association recognizes the value of peer review: “The peer review process is intended to balance physicians’ right to exercise medical judgment freely with the obligation to do so wisely and temperately.” [https://www.ama‐assn.org/delivering‐care/ethics/peer‐review‐due‐process]

#### 1.1.3 Accreditation and certification

Peer review is one approach to meeting Part IV: Assessment of Performance in Practice for the Maintenance of Certification (MOC) program for the American Board of Radiology (ABR), and this applies to therapeutic, diagnostic, and nuclear medical physics. Physicist peer review is an integral component of the American College of Radiology's Radiation Oncology Practice Accreditation (ROPA) as well as the Accreditation Program for Excellence (APEx) practice accreditation program from the American Society for Radiation Oncology (ASTRO). For accredited clinical programs, the most recent accreditation review may inform the physicist peer review process, thereby reducing the overall time commitment for both the reviewer and the incumbent physicist.

#### 1.1.4 Current status

It is recognized that peer review is more established in the therapeutic medical physics specialty than in the diagnostic and nuclear medicine specialties, with more than 15 years of experience since the publication of the TG‐103 report^4^ and extensive promotion of peer review through therapy practice accreditation programs. Furthermore, practice settings are more diverse in the imaging physics and nuclear medicine physics specialties, with broad utilization of contracted physics services, often coupled with a narrowly defined scope of those services and the attendant resource requirements. Given these realities, professional peer review should be the standard of practice in clinical therapeutic medical physics, whereas peer review in clinical diagnostic and nuclear medical physics will likely remain a distinct characteristic of an exceptional program for the foreseeable future.

#### 1.1.5 Alternative approaches to professional peer review

In some practice settings, it may not be feasible to engage an external reviewer for a formal peer review, and alternative approaches may be necessary to accomplish the core objective of providing a productive critique of the incumbent's clinical physics program with the aim of enhancing the program and the incumbent's professional practice. Any alternative peer review approach must use a process that encourages a collegial, peer to peer, non‐punitive review and allows for external guidance to the practice. The following are some considerations for alternative peer review approaches:

- Documented process: A document describing the alternative peer review process specific to the practice environment.
- Regular intradisciplinary meetings: Regularly scheduled meetings where all medical physicists in the practice are able to attend, contribute meeting topics and discuss relevant medical physics issues in a non‐punitive environment.
- Built in review of processes: This can be accomplished via cross‐coverage, rotation of responsibilities, audits of QA reports and/or patient charts, or other methods that cultivate a peer review of medical physics processes. 
- External review: Practice accreditation provides external review of a practice. In addition, a defined process for participation in national meetings (remotely or in person) and for returning knowledge to the group, and/or regularly scheduled journal club meetings to review recent publications or practice guidelines, can provide external perspectives to enable the incumbent physicist(s) to keep track of relevant developments in the profession.

### 1.2 Principles of constructive professional peer review

Peer review is distinct from other types of professional review, such as a compliance audit or an employment annual review. Its focus is on practice improvement, helping the incumbent to be more effective in their role. Peer review should comprise not only a review of professional practice, but should also be a critical assessment of the practice setting: Is there sufficient institutional support? Are appropriate tools and resources available? Is the workload appropriate for thoughtful and thorough work?

An approach focusing on systemic aspects of the work environment rather than strictly personal performance is termed “just culture,” one aspect of which recognizes that many errors are not the fault of the individual, but instead reveal weaknesses in the system in which the individual is working. According to the American College of Radiology, “A just culture is an environment in which errors and near‐miss events are evaluated in a deliberately nonpunitive framework, avoiding a culture of blame and responsibility and focusing instead on error prevention and fostering a culture of continuous quality improvement.^8^”

Deficiencies revealed by the peer review process may not necessarily be the fault of the incumbent. If the workload is excessive for one individual, it is easy for that individual to miss important aspects of practice. Therefore, the reviewer should carefully look for systemic causes rather than assuming the worst of the incumbent.

An effective peer reviewer remains acutely aware that the incumbent and their practice are the reason for the review process and avoids references to how the reviewer prefers to practice, unless such references are helpful in illustrating specific opportunities for improvement in the incumbent's practice setting. The reviewer should identify commendable aspects of the incumbent's practice and affirm these in the summary report.

### 1.3 Scope

Though therapeutic medical physics has a deeper history of peer review than does diagnostic medical physics and nuclear medicine physics, peer review can be of great benefit to all aspects of clinical medical physics. Therefore, the core recommendations in this document are intended to apply to any clinical physics peer review. The document was developed with external peer reviewers (having a different employer arrangement from the incumbent) as the primary focus. It is recognized that in larger group practices there may be other, equally effective, approaches to peer review.

In this document, the principles outlined above will be expanded and implemented. It will cover the process of conducting a peer review, from the initial contact to the final report. Specific applications for both therapy and diagnostic physicists will be presented. In the Appendix are included an example survey tool and sample reports.

Note that this is not intended to be a guide for self‐audit. While the desired result might be similar, such an audit requires a different approach and different tools.

## 2 ROLES AND RESPONSIBILITIES

### 2.1 Reviewer

The reviewer must be a Qualified Medical Physicist (QMP) as defined by the AAPM Position Statement^9^ and be a peer of the incumbent in the same specialty. Whenever possible, the reviewer should not be a manager, supervisor, or other superior to the incumbent as this may inhibit the peer review process. The reviewer should not have a close personal relationship with the incumbent, though it is recognized that this may be unavoidable in larger group settings.

It is the responsibility of the reviewer to ensure that the peer review is done in a way that encourages professional growth for the incumbent in a non‐punitive way.^10^ The reviewer must promote a confidential, safe environment in which the incumbent is able to speak freely about workplace challenges and best practices. The reviewer should not have any attachment to or investment in the results of the peer review. It is the responsibility of the reviewer to provide the results of the peer review in a meaningful and constructive manner both verbally in the exit interview and documented in a confidential peer review report.

### 2.2 Incumbent

The incumbent must be a practicing clinical medical physicist. It will benefit the peer review process if the incumbent is seeking to evaluate their clinical practice for potential improvement opportunities.

It is the responsibility of the incumbent to be a willing participant in the process with the intent of practice improvement. The incumbent must be honest with the reviewer and forthcoming about limitations and challenges in the workplace.^11^

### 2.3 Stakeholders

Stakeholders are individuals with a vested interest in the peer review process. These may include administrators, supervisors, managers, and department chairs.

The responsibility of the stakeholders is to recognize the importance of and support the peer review process as an opportunity for professional growth. The stakeholders must allow for a constructive and open process where the reviewer is able to peer review the incumbent without interference.

## 3 THE PEER REVIEW PROCESS

### 3.1 Introduction to the section and topics to be covered

In this section, we provide recommendations for the process of conducting peer reviews of clinical medical physicists. The primary focus of these recommendations is to ensure that the peer review is constructive for the incumbent physicist and disconnected from unrelated matters such as personnel decisions (e.g., annual performance evaluations). We provide recommendations for the frequency of review, the initiation of the review process, criteria for reviewer selection, the conduct of the peer review, reporting the findings of the review, and follow up.

### 3.2 Frequency of the review

Peer review should occur at least once every 36 months. However, additional reviews may be indicated for situations such as significant staffing changes in the practice, or the introduction of new technologies or clinical services. Such additional reviews are at the discretion of the incumbent physicist.

### 3.3 Initiation of the review

- Consistent with the goal of a peer review that is constructive for the incumbent physicist, we suggest that the most positive review environment is possible when the incumbent clinical physicist voluntarily initiates the review. We recognize that initiation by an employer does happen and is entirely appropriate in many situations. To ensure a constructive review when the employer initiates the review:
  1. The incumbent physicist should confirm that the reviewer's experience appropriately overlaps with the clinical scope of the incumbent's responsibilities, and the incumbent and reviewing physicists must manage any potential conflict of interest between the reviewing and incumbent physicists;
  2. The employer must verify that the reviewing physicist is a QMP in the specialty being reviewed, and must empower the reviewing physicist to use independent professional judgment;
  3. All parties (incumbent, stakeholders, and reviewer) should carefully review guidance documents related to the clinical physics peer review, such as this MPPG, to affirm their mutual understanding of the goals, expectations, and responsibilities.
- The reviewer must be appropriately compensated for the peer review services. We recognize the potential or perceived bias arising from any payer‐payee relationship. To address potential bias concerns, the employer must empower the reviewer to use independent professional judgment. The employer must not restrict the open communication between the incumbent and reviewer, and the reviewer must ensure that the incumbent receives a copy of the report prior to or at the same time as the employer. [Note: Reviewer compensation models may vary depending on whether the reviewer is external or internal.]

### 3.4 Selection of reviewer

- The reviewer must be a QMP according to the AAPM definition.
- The reviewer should be independent of the incumbent physicist, without a conflict of interest or commitment. Potential or perceived conflicts of interest or bias must be managed in accordance with the AAPM Code of Ethics^11^ and any other applicable codes of ethics (e.g., institutional). Avoidable conflicts include not choosing reviewers with the ability to directly affect employment, compensation, promotion, retention, tenure, or funding. Some potential conflicts of interest may be more nuanced. Former colleagues, mentors/mentees, advisors/trainees, and supervisors may have very relevant experience, but may not be entirely impartial. In a larger group practice, it may not be possible to avoid a current or prior professional relationship. Where such relationships exist between the reviewer and incumbent physicist, clear disclosure is appropriate.
- The reviewer should be experienced with the modalities to be reviewed. Further consideration should be given to potential reviewers who have served as surveyors for nationally recognized accreditation programs. The scope of the clinical physics program must be clearly communicated to the potential reviewer. If the incumbent physicist has concerns about a particular aspect of the physics program, the incumbent and potential reviewer should discuss prioritization of the planned review. Workflows and standard operating procedures may be reviewed remotely prior to the on‐site visit to be more efficient and allow for a cohesive review plan to be formulated prior to the on‐site visit. Prior to conducting peer reviews, the reviewer must become familiar with this MPPG and related examples and templates.
- An effective peer reviewer must exhibit a number of qualities and abilities that enable the individual to conduct the review in a professional and supportive manner. Examples of these qualities are listed in Table I.
- The reviewer and the incumbent's institution(s) should execute a formal written agreement describing the scope of the review, confidentiality, and other important legal considerations. The incumbent should consider alternating reviewers to provide fresh perspectives as the program evolves.

**TABLE 1:** Reviewer qualities.

Quality/Ability	Description
Adaptability	Recognize that different approaches by an incumbent physicist may yield similar results, and may be necessitated by factors such as staffing schedules, physician practice styles, and technology. Seek to understand the process sufficiently to assess whether appropriate practice standards are applied.
Collegial dialogue	Understand the incumbent's existing processes by listening to the incumbent explain the process, repeating back to the incumbent any steps that appear ambiguous.
Confidentiality	Adhere to the confidentiality requirements of the incumbent's clinic, and do not condition the review on access to clinic documentation beyond the scope and time window of the peer review.
Constructive feedback	Suggest potential improvements in the existing process and provide relevant references to help the incumbent improve their existing processes. As stated in the AAPM Code of Ethics,^11^ “The reviewer's primary professional obligation is to help the reviewed professional recognize how to improve their professional practice.”
Educational focus	Emphasize the learning aspect of the peer review process, establishing a low‐stakes “safe zone” to allow the incumbent to openly discuss areas of concern and to actively seek feedback to improve the practice.
Inquisitiveness	Be flexible in accommodating the explanations and reasoning of the incumbent, asking questions about the process followed in the clinic.
Professionalism	Recognize that practice patterns vary between clinics. Provide ample opportunity for the incumbent to explain the reason for any deviation from expected standards.
Safety	Approach any deviation from standard practice with a risk‐informed assessment.

### 3.5 Preparation

A successful peer review does not happen without careful planning. The agenda should be established, interviewees should be identified and the times for those interviews should be scheduled in advance. Good communication is essential to a rewarding peer review process.

Other important aspects of the peer review process are the support from administration for the actual activity and engaged staff who work to maintain a strong safety culture. A recent report on peer review in Radiation Oncology^12^ states: “Departmental leadership should emphasize the importance of peer review and encourage others to actively engage in quality and safety initiatives. Otherwise, a major possible pitfall is that peer review becomes an activity that needs to be checked off a to‐do list and staff are distracted and/or uninterested.” Administrators and staff must contribute to an environment where peer review is supportive and considered a part of routine good practice. The incumbent should be afforded appropriate time to prepare for the review, and the day of the on‐site review should be considered a professional development day without other scheduled tasks. In the event of unforeseen clinical priorities, the administration, incumbent and reviewer must coordinate to ensure that a constructive review can be conducted without compromising patient care. This could include rescheduling the time for the on‐site review.

The first step in the execution is to select the reviewer. Due to the invasive nature of a review, the incumbent and reviewer must have a professional rapport. The reviewer may therefore be a known colleague.

### 3.6 Planning phase

1. Early in the planning phase, the scope of the review must be established. Both parties must understand the expectations and agree on the clinical breadth. The parties must mutually decide if there are particular topics that should or should not be included. Note that this should be generally determined by the incumbent physicist and the reviewer, not by the clinic's administration, to ensure that it is a beneficial process for the incumbent.
2. The timeline should be established well before the peer review starts. Items that should be part of the timeline include a date by which the incumbent physicist is to provide preliminary documentation, the date of the review, the expected duration of the review, and the date by which the final report will be provided. It is possible that a review will be performed virtually, and this presents another layer of complexity. For virtual reviews, the technology to be used should be tested prior to the review date, and consideration should be given to providing a visual “tour” of the facility. In both cases, a clear agenda should be created, including who will be interviewed as part of the review process.
3. If available, previous peer review reports should be provided to the reviewer.
4. The reviewer should be aware of the incumbent's practice setting. A rural, solo practice has different needs from a large, urban practice. For example, information technology support and hospital radiation safety programs may be quite different. The reviewer should additionally be sensitive to special circumstances that might impact the incumbent's practice.
5. As the review will most likely include accessing patient records, the incumbent physicist should ensure that any institutional credentialing is completed before the reviewer comes on‐site or is granted remote access. An extremely important part of the review process is maintaining patient confidentiality consistent with the federal HIPAA regulations, as the reviewer will be functioning in the role of a “business associate” as defined under those regulations.

### 3.7 Review phase

1. The hours of operation of the site should be respected by the reviewer. The review should be kept within normal business hours unless it is specifically arranged otherwise prior to the visit. The review process can be stressful and exhausting for both the incumbent and the reviewer; keeping to normal hours with regular breaks will help to alleviate this issue.
2. The reviewer should be able to give their full attention to the peer review process without interruption. The review should be scheduled at a time that is mutually acceptable to both the incumbent physicist and the reviewer. The reviewer should never show up as a surprise. Instead, the review should be carefully planned with an agreed upon timeline.
3. While most facilities have adequate technology for the peer review, it will be necessary to ensure that the reviewer has adequate access. The reviewer should have easy access to the Internet. Logins to network drives, clinical software applications or other proprietary systems might be needed. When direct access to records or software applications for the reviewer cannot be obtained, the incumbent or a designee must assist the reviewer during the review. It may also be necessary to secure building access to facilitate the review.

## 4 PEER REVIEW

The review must include assessments of the available resources, the incumbent physicist's work product, and professional skills. Recommendations for assessment methodology are provided, along with some tools to facilitate the review process.

### 4.1 Available resources

1. The reviewer must determine whether instruments (appropriate to the clinical scope) are readily available, with instrument calibrations consistent with current AAPM recommendations and/or applicable regulations.
2. Staffing levels and schedules must be carefully assessed, with consideration for the scope of clinical services, frequency of special procedures requiring physicist support, and other physicist duties such as radiation safety and administrative tasks. The staffing assessment should consider support staff such as medical physicist assistants,^13^ medical dosimetrists, administrative support, and information technology.
3. Equipment access for quality control and quality assurance must be assessed, including flexibility of scheduling relative to the incumbent physicist's availability. This is particularly important when the physicist is responsible for multiple clinic locations or is contracted for less than full‐time coverage.

### 4.2 Work product

1. Core physics conventions and assumptions [such as those used for accelerator calibrations and brachytherapy source strength verification in radiation oncology, or the assumptions and correction factors used in peak skin dose calculations in imaging] must be clearly documented to ensure that other physicists can verify critical findings.
2. The reviewer must assess whether the machine and instrument QC records and templates are appropriate to the scope of the program, and whether the test methodology and action levels are appropriate and consistent with nationally accepted standards, AAPM task group reports, and Medical Physics Practice Guidelines. Key QC templates should be evaluated for accuracy and clarity.
3. The reviewer must assess whether clinical physics quality assurance (e.g., radiation oncology treatment plan reviews,^14^ fluoroscopy dose, and pregnant patient radiation safety evaluations) is appropriate to the clinical services being provided, whether the incumbent physicist has conducted risk assessments to justify the existing QA procedures, and whether appropriate tools exist to ensure that such QA is consistently performed.
4. The review must evaluate the program's safety culture and patient safety initiatives (e.g., incident learning, open communication, safety checklists).
5. The review must evaluate the incumbent's supervision of clinical operations and leadership in process improvement, such as overseeing the dosimetric planning process in therapy or overseeing the development and management of imaging protocols and patient shielding practices in imaging. For QC work delegated to support staff, the reviewer must ascertain that instructions and tolerances are unambiguous, that the results are reviewed and co‐signed by the incumbent physicist, and that the work is performed under appropriate supervision.^13^

### 4.3 Physicist skills

The reviewer must assess the incumbent physicist's professional skills relative to the requirements of the position. Examples of these skills are described in Table 2.

**TABLE 2:** Physicist skills, relative to the scope of the position.

Physicist skill	Description
Compliance	Evaluate the incumbent's compliance with accreditation standards and applicable regulations, and the quality of related documentation.
Professionalism, culture, and communication	Interview other members of the clinical team to assess how the incumbent interacts with other professionals and contributes to a constructive work environment.
Project management and introduction of new technology	If the incumbent has supported new machine installations, new facility construction, new technology, new processes, etc., such projects should be the basis for a discussion regarding the incumbent's approach to project management. In the absence of such recent projects, the reviewer should ask the incumbent to describe their approach and the institution's overall approach to the introduction of new technology or services.
Resource utilization	The incumbent should demonstrate awareness of the facility's resource utilization such as staff time, consumables, and contracted services, and should demonstrate a deliberative approach to ensuring that resources are focused on appropriate clinical and safety priorities.
Patient and staff safety	The incumbent should actively contribute to building and maintaining a safety culture, with collaborative efforts among the clinical team members to reach a shared understanding of the workflows and inherent risks, and structured analysis of incidents.
Radiation Safety	Evaluate the incumbent's radiation safety duties, if applicable, and determine if the program properly establishes radiation safety standards and complies with local and national regulations and standards. Verify that the incumbent is afforded appropriate time to complete these duties.
Teaching/training	Evaluate the incumbent's approach to and effectiveness in clinical teaching or training, if applicable.
Continuing education	Confirm that the incumbent has appropriate training in the technologies used at the facility, including regular participation in continuing education in accordance with the requirements of local policies, regulations, certification, and accreditation programs.
Career development	The incumbent should describe their professional aspirations and recent activities with regard to career development.

## 5 ASSESSMENT METHODOLOGY AND TOOLS

Several different approaches to evaluation are available, and different aspects of the review may lend themselves to different methods. Some aspects of the review may best be evaluated using a performance scale, such as 1−3 or “deficient,” “meets practice expectations,” and “exceeds expectations.” Others may be binary options: yes or no, for example, whether the incumbent has this piece of equipment or does this activity. For some topics, a subjective narrative approach may be most appropriate. Observations and suggestions are best communicated in the narrative form.

The reviewer should use a survey tool that covers all of the elements that are to be part of the review and incorporates the various evaluation techniques as appropriate. This will help to keep the review process on track and ensure that the review is complete and that nothing is overlooked. A comprehensive survey tool has been developed by the MPPG 15 task group and is available at https://www.aapm.org/pubs/MPPG.

## 6 ORAL EXIT SUMMARY

At the conclusion of the review process, the reviewer should provide an informal, oral summary of key observations to the incumbent. The purpose of this summary is to clarify any misinterpretations or omissions by the reviewer, and for the incumbent to provide relevant context to the reviewer's observations before the reviewer prepares the written report. In some cases, it may be appropriate to also seek clarification from other key professionals in the practice, such as the medical director or department administrator.

## 7 WRITTEN REPORT

The reviewer must provide a written report to the incumbent physicist within one month of the on‐site review. If other stakeholders request a report, a stakeholder report should be provided. A copy of this stakeholder report should also be provided to the incumbent physicist. The reports must be identified as “Privileged and Confidential Peer Review” to clearly express the confidential nature of the peer review for the purpose of continuing professional development. Examples are provided in the Appendix.

The incumbent physicist report should be organized into major and minor recommendations:

- Major recommendations are items that, in the reviewer's opinion, do not presently meet applicable regulations or minimum practice guidelines, or scenarios that could potentially result in harm to patients or staff. When appropriate, major recommendations should include a reference to relevant national guidance documents such as AAPM Medical Physics Practice Guidelines and ACR Technical Standards. [Major recommendations are expected to be rare in a well‐managed program.]
- Minor recommendations relate to items that have no impact on regulatory compliance or generally accepted guidelines but could enhance the physics program's productivity or level of organization/documentation.

The report should include a summary page that simplifies the recommendations in a straightforward manner, followed by detailed information gathered during the peer review process to elaborate on the recommendations. As a part of the summary, the reviewer should recommend follow‐up in accordance with their findings.

The stakeholder report (if provided) should be an executive summary with information that would be instructive to the stakeholders. Examples include staffing recommendations, equipment needs, or organizational suggestions. A general reference to the physics portion of the peer review is appropriate to include, provided it is productive and does not jeopardize the confidence of the peer‐to‐peer nature of the review. The overall intent of the stakeholder report is to demonstrate where the incumbent physicist may require additional support from the organization.

## 8 FOLLOW‐UP

Given the work that goes into performing the review and generating the final report, it behooves the incumbent and facility to address the recommendations in the report. It is suggested that a follow‐up plan be developed within one month of receiving the report, providing a path to be followed and time frames in which each follow‐up action should be completed.

Templates have been developed to assist with the review process and the generation of the peer review report(s). These templates are available for download from the JACMP article page under the “Supporting documentation” link.

## AUTHOR CONTRIBUTIONS

This guideline was reviewed and updated by Medical Physics Practice Guideline Task Group 358 of the Professional Council of the AAPM. Each author reviewed recent literature on the topic and offered opinions on and language for the guideline. They also reviewed and applied comments from the full AAPM membership to the document.

## CONFLICT OF INTEREST STATEMENT

The members of Medical Physics Practice Guideline 15.a: Peer Review in Clinical Physics (TG‐358) listed below attest that they have no potential Conflicts of Interest related to the subject matter or materials presented in this document.

Per H Halvorsen, MS, FAAPM, FACR, Chair

Alan H Baydush, PhD

Courtney R Buckey, PhD

Navneeth Hariharan, MEng

Mary Ann Keenan, DMP

Jeffrey P Limmer, MS, FAAPM

Kate E Lofton, MS

Robin A Miller, MS, FAAPM

Jeffrey M Moirano, MS

Joseph Och, MS, FAAPM

Douglas E Pfeiffer, MS, FAAPM, FACR

Nicholai Wingreen, AAPM Staff

## Supporting information

Example Peer Review Reports

Peer Review Report Template

Peer Review Data Collection Tool
