# Supplementary material for: AAPM Medical physics practice guideline 15.A: Peer review in clinical physics
Source: J Appl Clin Med Phys. 2023 Sep 14;24(10):e14151. doi: 10.1002/acm2.14151 (PMC10562014; doi:10.1002/acm2.14151)
Supplement: Supplementary file 1 — Example Peer Review Reports [file ACM2-24-e14151-s001.pdf]

APPENDIX

NOTE: The following examples are provided to illustrate the aforementioned guidelines for peer review. These are <u>examples only</u> and should not be directly applied to a clinical physics peer review. Each practice setting is unique and requires appropriate judgment by the reviewer as described in the guideline.

[Subsequent pages contain example reports for two scenarios for each specialty – a "clean" scenario and a "remedial" scenario (to illustrate how a reviewer can report on a deficient program in a constructive manner).]

24 November 2021

Dr. John Smith
Medical Director
Best RadOnc

Dear Dr. Smith,

Included is a summary of the clinical physics peer review report for Best RadOnc located at Xray Street in Nowhere, MA. The review was performed on 23 November 2021 with the on-site assistance of Dr. Incumbent and was conducted in accordance with the AAPM's Medical Physics Practice Guideline (MPPG) 15 for clinical physics peer reviews.

The MPPG 15 guideline defines two categories of recommendations. 'Major recommendations' are items that, in the reviewer's opinion, do not presently meet applicable regulations or generally accepted guidelines, or scenarios that could result in harm to patients or staff. 'Minor recommendations' relate to items that have no impact on regulatory compliance, generally accepted guidelines or safety, but could enhance the physics program's productivity or level of organization.

I'm pleased to report that the review found **no** deficiencies meeting the 'Major recommendations' category in MPPG 15.

I have provided Dr. Incumbent with a list of 'Minor recommendations'. Of these, I would like to bring your attention to the following recommendation as it reflects a need for support from the institution to the physics program:
Recommend an additional 0.5 FTE physics staffing to support program development given the rapid adoption of SRS-SBRT services, and additional instrumentation such as a dynamic phantom to support the same services.

If you have any questions related to this report, please contact me at your convenience.

Respectfully Submitted,

[Insert Scanned Signature]

Physicist Reviewer, MS, DABR
111-111-2222
reviewer@example.com

CONFIDENTIAL

24 November 2021

Dr. Incumbent
Best RadOnc
Nowhere, MA

Dear Dr. Incumbent,

Thank you for the courtesy extended to me during my visit to Best RadOnc on 23 November 2021 for the purpose of performing a professional peer review of your clinical physics program. Your program documentation is outstanding, which greatly facilitated the review process.

The review was conducted in accordance with the AAPM Medical Physics Practice Guideline 15 on clinical physics peer review. The enclosed report details the aspects of the clinical physics program that were reviewed and my findings for each component. A tour, review of quality management records and instrumentation, and conversations regarding institutional support and program needs were conducted while on site. As described in MPPG 15, the primary purpose of the peer review is to support your professional practice, and the review and resulting report were focused on that goal.

The MPPG 15 guideline defines two categories of recommendations. ‘Major recommendations’ are items that, in the reviewer’s opinion, do not presently meet applicable regulations or generally accepted guidelines, or scenarios that could result in harm to patients or staff. ‘Minor recommendations’ relate to items that have no impact on regulatory compliance, generally accepted guidelines or safety, but could enhance the physics program’s productivity or level of organization.

I’m pleased to report that the review found **no** deficiencies meeting the ‘Major recommendations’ category in MPPG 15.

The report provides a list of ‘Minor recommendations’ aimed at assisting you in further enhancing the clinical physics program.

I hope you find the review process, and the attached report, to be helpful to your practice. I’m available to clarify any aspect of the report.

Respectfully Submitted,

[Insert Scanned Signature]

Physicist Reviewer, MS, DABR
111-111-2222
reviewer@example.com

CONFIDENTIAL

General, Operations and Facility
The department is housed in an annex to the hospital constructed approximately 10 years ago, and houses a Varian TrueBeam accelerator, Philips Big Bore CT-simulator and Varian GammaMed HDR system. The center provides a typical range of external beam services as well as a moderately busy HDR service, and recently introduced SRS and SBRT services.

Staff Credentials, Coverage and Team Work
Two radiation oncologists share coverage of the clinic, each working 4 days per week. Dr. Incumbent works full time for the hospital as the solo physicist, with occasional access to a local physicist on a contract basis for coverage though the contractor's availability is inconsistent. A single dosimetrist provides treatment planning coverage, and the center employs 5 therapists including the chief therapist, and a single nurse. The team exhibits excellent and open communication and meets regularly to review patients' status.

State / Regulatory
The review found no items of regulatory non-compliance.

Radiation Safety / Radioactive Materials
Radioactive materials are appropriately surveyed on receipt, securely stored, and properly inventoried. The physicist conducts annual refresher safety training with all relevant staff.

Quality Management
The team meets quarterly to review the program's quality management and works collaboratively on identified priorities, but the quality management program has not been updated to reflect the recent addition of potentially high-risk procedures such as SRS and SBRT and does not participate in structured incident learning. The team should reassess the quality management program from a risk-informed perspective with special focus on the new SRS and SBRT services, consistent with the recommendations of the AAPM TG-100 report.

Major Equipment QA
The equipment QA program is well organized and documented and meets the traditional AAPM Task Group recommendations, but has not been updated to reflect the new services. Dr. Incumbent should review the program in light of the new SRS-SBRT services and recent AAPM guidelines in that regard, focusing on small-field and small-target treatments and motion management including gated treatment delivery.

Instrumentation
The center has appropriate instrumentation for key output calibrations and image quality assessments, but should acquire a dynamic phantom for validation of 4DCT and gated delivery, and should consider acquisition of a more suitable small-field dosimeter.

Special Procedures [If applicable]
The center has recently added SBRT and SRS services, including SBRT for oligometastatic disease and single-isocenter multi-target (SIMT) cranial SRS. The program should be updated to provide more robust procedures for evaluation of prior treatments and assessment of MR distortion and 6DOF alignment and their effects on accuracy for small off-center targets.

CONFIDENTIAL

Software and Networking QA
The treatment planning system QA program is well documented and generally consistent with MPPG 5, but should be enhanced to include a small-target scenario and off-center targets as seen with SIMT SRS. I would also recommend the creation of a suite of test plans for use during upgrade testing.

Patient-specific QA [If applicable]
The IMRT QA program is consistent with the TG-218 recommendations.

Patient Chart Reviews [If applicable]
10 charts were reviewed, including two HDR treatments, SRS and SBRT cases and several IMRT cases. Overall, the chart documentation is excellent and the physicist's review of all charts was consistent and timely. Dose objectives for plan evaluation are appropriate for each site and fractionation and consistent with current guidelines.

New Services (Planned and Actual)
The team has done a commendable job managing the rapid adoption of SRS and SBRT services without any adjustment in staffing levels. Given the lean staffing model, it's understandable that they have had little time for program evaluation and quality management program updates. The institution should reassess the staffing levels in physics, therapy and dosimetry to ensure that the team has the bandwidth for meaningful program quality reviews and improvements.

Professionalism and Professional Development
Dr. Incumbent is seen by the entire clinical team as a leader and is viewed by therapists and physicians as a natural resource for troubleshooting and advice, always responding to such inquiries in a respectful and welcoming manner and providing clear explanations of complex concepts. Given the lean staffing model, she has understandably not had sufficient bandwidth to focus on professional development in recent years nor to undertake a comprehensive program review. The institution would be well served by providing additional physics coverage to enable Dr. Incumbent to invest the appropriate time and effort on these priorities, and I'm confident she has the professional skills to succeed when given the appropriate resources.

CONFIDENTIAL

Conclusions/Recommendations/Comments

The clinical team displays a high level of cohesion and effective communication and is well positioned to focus on quality improvements provided the institution provides the resources to enable the team to invest the time necessary for such review and process improvements.

Based on the review, consistent with MPPG 15, I provide the following recommendations with the goal of supporting your professional practice and professional development:

<u>Minor Recommendations & Comments</u>

1. Reassess the quality management program with a focus on risk-informed analysis, consistent with the recommendations of TG-100 with particular emphasis on the new SRS and SBRT services.
2. Validate the 4DCT and gated treatment delivery accuracy once a dynamic phantom is available.
3. Add a small-target treatment scenario to the annual TPS QA program, ideally in the form of a full E2E process validation consistent with the MPPG 9 recommendations.
4. Assess the 3D MR distortion with the “SRS scan protocol” on the MR scanner(s) used for SRS scans, at a minimum using an E2E service such as RT-Safe, or with a dedicated MR distortion phantom.

Thank you again for your support of this peer review process.

Respectfully Submitted,

Physicist Reviewer, MS, DABR
24 November 2021

CONFIDENTIAL

Clinical Physics Peer Review
Site Data

**Date of review**	23-Nov-21
**Date of previous Peer Review**	N/A
**Physicist Peer Reviewer**	Physicist Reviewer

Review previous report if applicable

**Site name**	Best RadOnc
**Address**	1 Xray Street
**City**	Nowhere
**State**	MA
**Zip**	11111
**Phone**	111-22-3333
**Incumbent physicist(s) reviewed:**	**Dr. Incumbent**

**Staffing** (add rows if necessary)	**Comments**	**Certified?**	**FTE**
**Physician(s)**		ABR	1.0
**RSO**		Radiologist	0.1
**Physicist - Chief**		ABR	1.0
**Physicist(s) - Staff**			
**Physicist(s) - Consultant**		ABR	0.1
**Medical Physicist Assistant(s)**			
Other staff (explain in Comments)			
**Radiation Oncology staff:**			
Dosimetrist - Sr / Lead			
Dosimetrist - Staff		CMD	1.0
Chief Therapist		RTT	1.0
Radiation Therapist - Sr / Lead			
Radiation Therapist - Staff		RTT	4.0
Oncology Nurse(s)		OCN	1.0
**Imaging / Nuclear Medicine staff:**			
Mammo supervisor			
CT supervisor			
Radiography supervisor			
MRI supervisor			
Nuclear Medicine supervisor			
Imaging Nursing supervisor			
IR supervisor			
Ultrasound supervisor			
Department director			
Department medical director			

			**Comments**
**Clinic type:**	e.g. Free standing or Hospital, JV<br>Notes: Multiple locations, etc	Hospital, single location	
**Regional Affiliation Structure?**		None	
**Academic affiliation?**		None	
**Cooperative Clinical Trails?**		No	

**Accreditation**	
Accrediting body used	**ACR**

Clinical Physics Peer Review

Facility Services Overview - RADIATION ONCOLOGY

**Date:**	23-Nov-21
**Reviewer:**	Physicist Reviewer

Best RadOnc

Clinical services supported by physicist, and approximate annual caseload:

Service	Sub-category	# annually	Comments
External beam therapy	2D conventional	50	
	3DCRT	150	
	IMRT/VMAT	300	
	Stereotactic Body Radiotherapy (SBRT)	25	
	Stereotactic radiosurgery (SRS)	15	
	Image-Guided Radiotherapy (IGRT)	400	CBCT
	Respiratory Gating	10	
	Surface-Guided Radiotherapy (SGRT)		
	Total body irradiation (TBI) / Total Skin (TSE)		
Brachytherapy	Prostate seed implants		
	HDR	30	Prostate, GYN
	IVBT		
	IORT		
Radiopharmaceutical therapy	[Type, Sm 153, etc]		
Other (describe)			

Other services supported or provided by physicist:

Service	Detail		Comments
Imaging physics / coordination	Ultrasound (eg, for brachytherapy guidance)		TRUS
	CT imaging quality / dose evaluations		Annual constancy evals
	MR distortion assessment (eg for SRS)		Coordinate with imaging physicist
Radiation safety	Number of radioactive materials licenses:	1	
	Serve as RSO?	No	
Computer support	Number of workstations	20	
	Type of support: networking, applications?		Applications
Administrative	Employees supervised	1	
	Responsible for physics billing policies/audits?	Yes	
	Teaching responsibilities?	No	

Clinical Physics Peer Review
Equipment and Instrumentation - RADIATION ONCOLOGY

Best RadOnc

**Date:**	23-Nov-21
**Reviewer:**	Physicist Reviewer

Clinical Equipment: Add lines if needed

Category	Manufacturer	Model	S/N	Install Date	Comments (IGRT, MLC, EPID, VMAT, robotics, #CT
Linear accelerator 1	Varian	TrueBeam	2199	10/15/15	6DOF, HDMLC
Linear accelerator 2					
CT-Simulator	Philips	BBB	1600	06/01/11	16 slice, 4DCT
Other dedicated imaging					
HDR remote afterloader	Varian	GammaMed	64A124	06/01/11	
Stereotactic radiosurgery					
Other treatment systems					
Independent X-ray system (e.g. Brainlab)					
SGRT					
Respiratory Gating	Varian	RPM			
Other					
**Planning-related software systems***	**Manufacturer**	**Model**	**Software version**	**Install Date**	**Options (Deformable registration, biological, auto-contouring, auto-planning etc)**
System 1	Varian	Eclipse	16.1		Deformable, RapidPlan
System 2	LifeLine	RadCalc	6.3.4		Brachy, ROI
System 3					
System 4					
System 5					
	**Manufacturer**	**Model**	**Software**	**Install date**	**Describe level of electronic records**
Record & verify system	**Varian**	**Aria**	**16.1**		**Full EMR**
QA Database	ImageOwl	TotalQA			All electronic

Instrumentation

Category	Manufacturer	Model	Type	Last cal date	Comments
Ionization chamber 1	Std Imaging	A12	Farmer	07/11/21	
Ionization chamber 2	Std Imaging	A12	Farmer	07/15/21	Indirect
Ionization chamber 3					
Small-field detector 1	Sun Nuclear	EDGE	Diode		
Small-field detector 2					
Electrometer 1	PTW	UniDose		07/11/21	
Electrometer 2	Std Imaging	Max4000		06/15/20	
Well Chamber	Std Imaging	HDR1000+	Well	06/15/20	
Daily QA device	Sun Nuclear	DQA3			
IMRT QA device / system	Sun Nuclear	MapCheck2			Also portal dosimetry
3D Scanning System	PTW	BeamScan			
1D Scanning System	Sun Nuclear	1D tank			
Multidetector array					
Film dosimetry					
Calibration phantom	Gammex SNC	Solid Water			
Dynamic phantom					None - needed
Survey Meter	Ludlum	Model 3			
In-vivo dosimetry	Landauer	nanoDot	OSL		QC follows TG-191
Special-purpose phantoms					
Thermometer(s) (quantity)		Alcohol column			Two identical, intercompared
Barometer(s) (quantity)	NovaLynx	M202	Digital		Compared to NWS local station
Other phantoms					

Comments:
Describe anything about the facility that is not properly conveyed in the spaces above.
Also, *any specific aspects of the clinical physics program that the on-site physicist wishes to have the reviewer focus on* in addition to the core review components.

Clinical Physics Peer Review

Program Checklist - RADIATION ONCOLOGY

Date: 23-Nov-21
Reviewer: Physicist Reviewer

Best RadOnc

Place an "x" in the appropriate column

N/A	Yes	Conditional	No		COMMENTS
**General / Global**					
	x			Follow relevant ASTRO/ACR Practice Parameters and Technical Standards	
	x			Follow relevant AAPM TG Report & MPPG recommendations	
		x		Quality management program is appropriately comprehensive and risk-informed, and the center participates in structured Incident Learning	*Overall good QM program, but have not yet attempted to adopt TG-100 methodology for risk analysis and do not participate in Incident Learning*
	x			Physician and Physicist supervision is appropriate to scope of services	
		x		The physicist has considered "disaster recovery" options and has appropriate data backup / redundancy measures in place to enable the safe resumption of services after a significant disruption (computer networks, physical facility etc)	*Some critical files are backed up, but many are not, and backups are on a network share. Recommend a written inventory of critical files, and an off-line storage less susceptible to being compromised*
				Describe the IT network security/rights environment and the impact on the physicist's work environment (additional tasks, restricted ability to install and use specialty software etc)	*Physicist does not have local admin rights on workstations, and there is no dedicated IT liaison, which complicates installation and updates of oncology software applications.*
**State / Regulatory**					
	x			Compliant with applicable state and federal regulatory requirements.	
**Staffing Education, Credentials**					
				See Site Information tab	
	x			Appropriate reporting structure	
		x		Adequate staffing levels	*Physics staffing insufficient for new program development*
		x		Appropriate competency / credentialing procedures	*No formal process for on-boarding new staff*
**Radiation Safety / Radioactive Materials**					
	x			Radiation safety inservices are provided to all relevant staff annually	
	x			Package surveys/wipe tests are performed, and appropriate staff are trained according to	
	x			Radioactive materials are properly secured and inventoried	
**Quality Improvement**					
x				Review recommendations and action plans from any previous Peer Review. Include any unresolved issues in the current report.	
	x			Formal quality assurance & improvement program should include: chart rounds, retrospective chart audits, and morbidity & mortality reviews	
	x			Evidence that the physicist participates in QA meetings and presents documentation of QA activities	
**Major Equipment QA**					
				**External Beam Treatment Machines**	
	x			Commissioning report is available and appropriate in scope	
	x			Protocols for calibrating treatment machines follow those protocols currently published by the AAPM and adhere to state and federal guidelines.	
	x			%DD(10cm) values used for calibration factors are reasonable	
	x			An independent check of the output of each beam is performed annually (either mailed OSLD service or independent physicist check)	
		x		Monthly QA program is appropriate to the clinical utilization	*No test of gated delivery*
	x			Minimum two Radiation Therapists per machine while treating	
	x			Service and maintenance logs, and machine problem logs are consistently updated and reviewed	
				**Simulators (CT, x-Ray, PET-CT)**	
	x			Commissioning Report is available and appropriate in scope	
	x			Monthly QA program is appropriate to the clinical utilization	
**Instrumentation**					
	x			Primary instrumentation has current ADCL calibration	
	x			Secondary instruments are appropriately cross calibrated / intercompared	
	x			Barometers/thermometers are intercompared regularly	
	x			Daily QA devices are baselined against the primary instrument on a regular basis (at least annually and after any instrument repair)	
		x		Appropriate phantoms are available to test all utilized modalities (e.g. 4DCT, gated delivery, cranial SRS, SGRT, OBI/CBCT image quality and dose)	*No dynamic phantom*
**Brachytherapy**					
				**General**	
	x			Time out to verify patient identity and treatment site location	
	x			Physician and Physicist supervision during treatment	
				**HDR**	
	x			Appropriate QA program and source-strength validation methods	
	x			Appropriate safety program (area monitors, pre- and post-treatment surveys, emergency refresher training etc)	
				**LDR**	
	x			Sealed source brachytherapy data complies with standards (e.g. TG43 update (TG43U1) consensus data)	
**TPS and IMRT QA**					
				**Treatment Planning Systems (External Beam and Brachytherapy)**	
	x			Treatment planning computer model must be verified using actual beam data measured by the qualified medical physicist on the same treatment unit for which the model will be	
	x			TPS commissioning report is available and consistent with MPPG 5 recommendations	
				**IMRT**	
	x			Appropriate site-specific and fractionation-specific dose objectives	
	x			IMRT QA methods and passing criteria are consistent with TG-218 recommendations	

**SRS/SBRT**					
		x		Commissioning report is available showing relevant end-to-end process validations and clear description of overall process accuracy and any limitations	*No formal commissioning report (but comprehensive physicist records)*
		x		QA program is consistent with MPPG 9	*Small-field MLC aperture test*
	x			Physician and Physicist supervision consistent with TG-101 and ACR Practice Parameter recommendations	
**Clinical program supervision**					
	x			Physics contributes to SOP development and process evalutions and improvement, including simulation and treatment delivery procedures	
		x		Physicist support for and supervision of the treatment planning process is consistent with the ACR-AAPM Technical Standard for External Beam Treatment Planning	*Physicist performs thorough and timely plan checks but is not actively engaged in developing guidance for the planning process*
	x			Physics reviews of treatment plans are consistent with the TG-275 and MPPG 11 recommendations	

Clinical Physics Peer Review

Chart Review Checklist - RADIATION ONCOLOGY

Date: 23-Nov-21
Reviewer: Physicist Reviewer

Best RadOnc

Use the following convention when scoring each chart item:
N/A, Yes, Conditional, No

Select charts to be representative of the patient case mix, physicians, and treatment modalities at the center. For a single-modality, single-physician practice, 5 charts may be sufficient, whereas 10 or more charts may be appropriate for a practice with multiple physicians and complex case mix and treatment modality profile.

General for all charts	
**Treatment planning system (s)**	
Application, version	Eclipse, 16.1
Homogeneity Correction used	Yes
Second-check calculation method	RadCalc
**Record and Verify:**	
Application, version	Aria, 16.1
**IMRT QA Equipment / Procedures:**	
QA Device Used	MapCheck
Passing criteria	>95%, 2mm, 3%, true composite
**In-vivo dosimetry**	
System(s) used	OSL
Conditions for in-vivo dosimetry	Specific MD request
**Comments**	

Individual Treatment charts	Chart 1	2	3	4	5	6	7	8	9	10
Patient key *(clinic should maintain a table linking the key to the actual medical record number for potential follow-up on recommendations)*	PR1	PR2	PR3	PR4	PR5	PR6	PR7	PR8	PR9	PR10
Treatment site / Dx	Pelvis / Cervix	Prostate	H&N	Lung	Cranial mets	Esophagus	Breast - L	Prostate	Vaginal cuff	Breast - R
Type of plan: IMRT/3D/SRS/e-/HDR	IMRT/VMAT	HDR and IMRT	IMRT	SBRT	SRS	IMRT	3D/e-	IMRT	HDR	3D
MD reviewed / signed plan prior to tx	Yes	Yes	Yes	Yes	Yes	Yes	Yes	Yes	Yes	Yes
MD Rx is complete and agrees with plan**	Yes	Yes	Yes	Yes	Yes	Yes	Yes	Yes	Yes	Yes
Treatment plan documentation appropriate and clear	Yes	Yes	Yes	Yes	Yes	Yes	Yes	Yes	Yes	Yes
Setup information complete and clear, including photos**	No photos of tattoos	Yes	Yes	Yes	Yes	Yes	e- CD setup photos are	Yes	Yes	Yes
Initial physics plan review completed prior to first tx	Yes	Yes	Yes	Yes	Yes	Yes	Yes	Yes	Yes	Yes
Physics chart review & frequency are appropriate**	Yes	Yes	Yes	Yes	Yes	Yes	Yes	Yes	Yes	Yes
Therapists performed initial chart review prior to first tx	Yes	Yes	Yes	Yes	Yes	Yes	Yes	Yes	Yes	Yes
**Comments** (incl. evidence of strong safety culture)	*Clearance checks performed early in planning process*				*SIMT*			*20 fx*	*Monotherapy*	*Hypofx no CD*

Simu	Chart 1	2	3	4	5	6	7	8	9	10
Simulation documented incl. scan range, immobilization etc	Yes	Yes	Yes	Yes	Yes	Yes	Yes	Yes	Yes	Yes
Motion management documented	N/A	N/A	N/A	Yes	N/A	Yes	Yes	N/A	N/A	N/A
**Comments**				*4D ITV*	*1mm slices*	*4D ITV*	*DIBH*			

Elect	Chart 1	2	3	4	5	6	7	8	9	10
Appropriate distance factors / cutout factors used	N/A	N/A	N/A	N/A	N/A	N/A	Yes	N/A	N/A	N/A
**Comments**							110 SSD			

IMRT	Chart 1	2	3	4	5	6	7	8	9	10
QA complete / passed prior to first tx	Yes	Yes	Yes	Yes	Yes	Yes	N/A	Yes	N/A	N/A
QA passing criteria documented	Yes	Yes	Yes	Yes	Yes	Yes		Yes		
MD's dose objectives and actual plan values are clearly	Yes	Yes	Yes	Yes	Yes	Yes		Yes		
**Comments**								PROFIT criteria		

**IGRT/SGRT**	**Chart 1**	**2**	**3**	**4**	**5**	**6**	**7**	**8**	**9**	**10**
Type used	CBCT	CBCT	CBCT	CBCT	CBCT	CBCT	MV portal	CBCT	N/A	N/A
Frequency	Daily	Daily	Daily	Daily	Daily	Daily	Daily	Daily		
Daily shifts and documentation	Yes	Yes	Yes	Yes	Yes	Yes	Yes	Yes		
**Comments**					6DOF					

**SRS / SBRT**	**Chart 1**	**2**	**3**	**4**	**5**	**6**	**7**	**8**	**9**	**10**
Appropriate pre treatment checks and documentation	N/A	N/A	N/A	Yes	Yes	N/A	N/A	N/A	N/A	N/A
Prior treatment assessment if applicable				N/A	Yes					
Plan evaluation metrics appropriate (eg CI, R50 etc)				Yes	Yes					
Day-of-tx machine QA (eg Winston-Lutz) performed				Yes	Yes					
Physicist and physician supervision appropriate				Yes	Yes					
**Comments**					7 cm off axis, no extra PTV margin					

**Brachytherapy**	**Chart 1**	**2**	**3**	**4**	**5**	**6**	**7**	**8**	**9**	**10**
Site Identified	N/A	Yes	N/A	N/A	N/A	N/A	N/A	N/A	Yes	N/A
Written Directive prior to administration		Yes							Yes	
MD approved plan		Yes							Yes	
Plan checklist / 2nd-check calculation completed		Yes							Yes	
Applicator verification		Yes							Yes	
Time Out		Yes							Yes	
Daily system QA performed		Yes							Yes	
Patient tx checklist complete for each fraction		Yes							Yes	
Survey performed and documented pre and post tx		Yes							Yes	
AU present during tx		Yes							Yes	
AMP present during tx		Yes							Yes	
Complete tx documented		Yes							Yes	
documentation		N/A							N/A	
**Comments**										

Clinical Physics Peer Review

Programmatic questions - RADIATION ONCOLOGY

Best RadOnc

**Date:**	23-Nov-21
**Reviewer:**	Physicist Reviewer

To whom does the physicist report directly?	x	Medical Director	
	x	Radiation Oncology Manager	
		Executive Level Administrator	
		Other:	
Contract Physicist?	No		
Do the dosimetrist(s) report directly to the physicist?	Yes		
Does the physicst have a formal role in the facility's budgeting and technology selection processes?	Yes		
Does the present physicist coverage provide adequate time during normal clinic hours for the physicist to consult with the treatment team regarding specific patient issues?	Yes		
Does the present physicist coverage provide adequate time during normal clinic hours for the physicist to plan and prepare for the implementation of new services?	No		
Is there adequate coverage during a physicist absence?	Contracted local physicist - inconsistent availability		
Are any new clinical physics services planned for the coming year?	Increased use of SIMT SRS and SBRT for oligomets, including new treatment sites		
Does the RadOnc practice participate in structured incident learning? Is prospective risk analysis part of the quality management program, particularly with respect to introduction of new services?	No (both)		
Does the facility provide adequate physics instrumentation, machine access and resources to support the facility's clinical service?	Marginally; need dynamic phantom and more robust physics staffing		
Describe the machine service/support arrangements (hardware/software support, in-house engineers/biomed roles if applicable)	OEM service contracts, no in-house engineer		
Any other issues?			

24 November 2021

Dr. John Smith
Medical Director
Best RadOnc

Dear Dr. Smith,

Included is a summary of the clinical physics peer review report for Best RadOnc located at Xray Street in Nowhere, MA. The review was performed on 23 November 2021 with the on-site assistance of Dr. Incumbent and was conducted in accordance with the AAPM's Medical Physics Practice Guideline (MPPG) 15 for clinical physics peer reviews.

The MPPG 15 guideline defines two categories of recommendations. 'Major recommendations' are items that, in the reviewer's opinion, do not presently meet applicable regulations or generally accepted guidelines, or scenarios that could result in harm to patients or staff. 'Minor recommendations' relate to items that have no impact on regulatory compliance, generally accepted guidelines or safety, but could enhance the physics program's productivity or level of organization.

My review identified the following item meriting focused remedial attention:
The recent commissioning of SRS-SBRT treatment modalities was performed with a Farmer ionization chamber, which is not a suitable detector for characterizing small fields and could result in non-negligible beam modeling errors. The center should acquire a suitable small-field detector as soon as possible, and small-field measurements should be re-acquired followed by validation of the beam model and model revision if warranted. I advise suspension of the SRS-SBRT service until this remedial work is completed.

I have also provided Dr. Incumbent with a list of 'Minor recommendations'. Of these, I would like to bring your attention to the following recommendation as it reflects a need for support from the institution to the physics program:
Recommend an additional 0.5 FTE physics staffing to support program development given the rapid adoption of SRS-SBRT services, and additional instrumentation such as a dynamic phantom to support the same services.

If you have any questions related to this report, please contact me at your convenience.

Respectfully Submitted,

[Insert Scanned Signature]

Physicist Reviewer, MS, DABR
111-111-2222
reviewer@example.com

CONFIDENTIAL

24 November 2021

Dr. Incumbent
Best RadOnc
Nowhere, MA

Dear Dr. Incumbent,

Thank you for the courtesy extended to me during my visit to Best RadOnc on 23 November 2021 for the purpose of performing a professional peer review of your clinical physics program. Your program documentation is outstanding, which greatly facilitated the review process.

The review was conducted in accordance with the AAPM Medical Physics Practice Guideline 15 on clinical physics peer review. The enclosed report details the aspects of the clinical physics program that were reviewed and my findings for each component. A tour, review of quality management records and instrumentation, and conversations regarding institutional support and program needs were conducted while on site. As described in MPPG 15, the primary purpose of the peer review is to support your professional practice, and the review and resulting report were focused on that goal.

The MPPG 15 guideline defines two categories of recommendations. 'Major recommendations' are items that, in the reviewer's opinion, do not presently meet applicable regulations or generally accepted guidelines, or scenarios that could result in harm to patients or staff. 'Minor recommendations' relate to items that have no impact on regulatory compliance, generally accepted guidelines or safety, but could enhance the physics program's productivity or level of organization.

My review identified the following item meriting focused remedial attention:
The recent commissioning of SRS-SBRT treatment modalities was performed with a Farmer ionization chamber, which is not a suitable detector for characterizing small fields and could result in non-negligible beam modeling errors. The center should acquire a suitable small-field detector as soon as possible, and small-field measurements should be re-acquired followed by validation of the beam model and model revision if warranted. I advise suspension of the SRS-SBRT service until this remedial work is completed.

The report provides a list of 'Minor recommendations' aimed at assisting you in further enhancing the clinical physics program.

I hope you find the review process, and the attached report, to be helpful to your practice. I'm available to clarify any aspect of the report.

Respectfully Submitted,

[Insert Scanned Signature]

Physicist Reviewer, MS, DABR
111-111-2222
reviewer@example.com

CONFIDENTIAL

General, Operations and Facility
The department is housed in an annex to the hospital constructed approximately 10 years ago, and houses a Varian TrueBeam accelerator, Philips Big Bore CT-simulator and Varian GammaMed HDR system. The center provides a typical range of external beam services as well as a moderately busy HDR service, and recently introduced SRS and SBRT services.

Staff Credentials, Coverage and Team Work
Two radiation oncologists share coverage of the clinic, each working 4 days per week. Dr. Incumbent works full time for the hospital as the solo physicist, with occasional access to a local physicist on a contract basis for coverage though the contractor's availability is inconsistent. A single dosimetrist provides treatment planning coverage, and the center employs 5 therapists including the chief therapist, and a single nurse. The team exhibits excellent and open communication and meets regularly to review patients' status.

State / Regulatory
The review found no items of regulatory non-compliance.

Radiation Safety / Radioactive Materials
Radioactive materials are appropriately surveyed on receipt, securely stored, and properly inventoried. The physicist conducts annual refresher safety training with all relevant staff.

Quality Management
The team meets quarterly to review the program's quality management and works collaboratively on identified priorities, but the quality management program has not been updated to reflect the recent addition of potentially high-risk procedures such as SRS and SBRT and does not participate in structured incident learning. The team should reassess the quality management program from a risk-informed perspective with special focus on the new SRS and SBRT services, consistent with the recommendations of the AAPM TG-100 report.

Major Equipment QA
The equipment QA program is well organized and documented and meets the traditional AAPM Task Group recommendations, but has not been updated to reflect the new services. Dr. Incumbent should review the program in light of the new SRS-SBRT services and recent AAPM guidelines in that regard, focusing on small-field and small-target treatments and motion management including gated treatment delivery.

Instrumentation
The center has appropriate instrumentation for key output calibrations and image quality assessments, but lacks a suitable small-field detector. Given the introduction of SRS-SBRT services, this should be remedied immediately. The center should also acquire a dynamic phantom for validation of 4DCT and gated delivery.

CONFIDENTIAL

Special Procedures [If applicable]

The center has recently added SBRT and SRS services, including SBRT for oligometastatic disease and single-isocenter multi-target (SIMT) cranial SRS. Of concern, a Farmer chamber was used for all beam data collection as well as model validation for SRS-SBRT. Farmer chambers are not suitable for small-field dosimetry. The small-field measurements must be repeated as soon as a suitable small-field detector is available, and the beam model must be adjusted as needed and re-validated. Given the potential dose modeling errors, I would strongly advise suspension of SRS-SBRT treatments until this can be rectified. The program should also be updated to provide more robust procedures for evaluation of prior treatments and assessment of MR distortion and 6DOF alignment and their effects on accuracy for small off-center targets.

Software and Networking QA

The treatment planning system QA program is well documented and generally consistent with MPPG 5, with the notable exception of the small-field beam modeling and validation as described above. The routine QA program should be enhanced to include a small-target scenario and off-center targets as seen with SIMT SRS. I would also recommend the creation of a suite of test plans for use during upgrade testing.

Patient-specific QA [If applicable]

The IMRT QA program is consistent with the TG-218 recommendations.

Patient Chart Reviews [If applicable]

10 charts were reviewed, including two HDR treatments, SRS and SBRT cases and several IMRT cases. Overall, the chart documentation is excellent and the physicist's review of all charts was consistent and timely. Dose objectives for plan evaluation are appropriate for each site and fractionation and consistent with current guidelines.

New Services (Planned and Actual)

The team has managed the rapid adoption of SRS and SBRT services without any adjustment in staffing levels. Given the lean staffing model, it's understandable that they have had little time for program evaluation and quality management program updates, and the lack of awareness of suitable small-field detectors is an example of the consequences of insufficient resources. The institution should reassess the staffing levels in physics, therapy and dosimetry to ensure that the team has the bandwidth for meaningful program quality reviews and improvements.

Professionalism and Professional Development

Dr. Incumbent is seen by the entire clinical team as a leader and is viewed by therapists and physicians as a natural resource for troubleshooting and advice, always responding to such inquiries in a respectful and welcoming manner and providing clear explanations of complex concepts. Given the lean staffing model, she has understandably not had sufficient bandwidth to focus on professional development in recent years nor to undertake a comprehensive program review. The institution would be well served by providing additional physics coverage to enable Dr. Incumbent to invest the appropriate time and effort on these priorities.

CONFIDENTIAL

Conclusions/Recommendations/Comments

The clinical team displays a high level of cohesion and effective communication and is well positioned to focus on quality improvements provided the institution provides the resources to enable the team to invest the time necessary for such review and process improvements.

Based on the review, consistent with MPPG 15, I provide the following recommendations with the goal of supporting your professional practice and professional development:

Major recommendation

Acquire a suitable small-field detector as soon as possible, and repeat the small-field measurements followed by validation (and revision as needed) of the beam model. Given the potential errors from small-field beam modeling based on Farmer chamber measured data, I strongly advise suspension of the SRS-SBRT service until the small-field modeling and validation is completed.

Minor Recommendations & Comments

1. Reassess the quality management program with a focus on risk-informed analysis, consistent with the recommendations of TG-100 with particular emphasis on the new SRS and SBRT services.
2. Validate the 4DCT and gated treatment delivery accuracy once a dynamic phantom is available.
3. Add a small-target treatment scenario to the annual TPS QA program, ideally in the form of a full E2E process validation consistent with the MPPG 9 recommendations.
4. Assess the 3D MR distortion with the "SRS scan protocol" on the MR scanner(s) used for SRS scans, at a minimum using an E2E service such as RT-Safe, or with a dedicated MR distortion phantom.

Thank you again for your support of this peer review process.

Respectfully Submitted,

Physicist Reviewer, MS, DABR
24 November 2021

CONFIDENTIAL

Clinical Physics Peer Review
Site Data

**Date of review**	23-Nov-21
**Date of previous Peer Review**	N/A
**Physicist Peer Reviewer**	Physicist Reviewer

Review previous report if applicable

**Site name**	Best RadOnc
**Address**	1 Xray Street
**City**	Nowhere
**State**	MA
**Zip**	11111
**Phone**	111-22-3333
**Incumbent physicist(s) reviewed:**	**Dr. Incumbent**

**Staffing** (add rows if necessary)	**Comments**	**Certified?**	**FTE**
**Physician(s)**		ABR	1.0
**RSO**		Radiologist	0.1
**Physicist - Chief**		ABR	1.0
**Physicist(s) - Staff**			
**Physicist(s) - Consultant**		ABR	0.1
**Medical Physicist Assistant(s)**			
Other staff (explain in Comments)			
**Radiation Oncology staff:**			
Dosimetrist - Sr / Lead			
Dosimetrist - Staff		CMD	1.0
Chief Therapist		RTT	1.0
Radiation Therapist - Sr / Lead			
Radiation Therapist - Staff		RTT	4.0
Oncology Nurse(s)		OCN	1.0
**Imaging / Nuclear Medicine staff:**			
Mammo supervisor			
CT supervisor			
Radiography supervisor			
MRI supervisor			
Nuclear Medicine supervisor			
Imaging Nursing supervisor			
IR supervisor			
Ultrasound supervisor			
Department director			
Department medical director			

			**Comments**
**Clinic type:**	e.g. Free standing or Hospital, JV<br>Notes: Multiple locations, etc	Hospital, single location	
**Regional Affiliation Structure?**		None	
**Academic affiliation?**		None	
**Cooperative Clinical Trails?**		No	

**Accreditation**	
Accrediting body used	**ACR**

Clinical Physics Peer Review

Facility Services Overview - RADIATION ONCOLOGY

**Date:**	23-Nov-21
**Reviewer:**	Physicist Reviewer

Best RadOnc

Clinical services supported by physicist, and approximate annual caseload:

Service	Sub-category	# annually	Comments
External beam therapy	2D conventional	50	
	3DCRT	150	
	IMRT/VMAT	300	
	Stereotactic Body Radiotherapy (SBRT)	25	
	Stereotactic radiosurgery (SRS)	15	
	Image-Guided Radiotherapy (IGRT)	400	CBCT
	Respiratory Gating	10	
	Surface-Guided Radiotherapy (SGRT)		
	Total body irradiation (TBI) / Total Skin (TSE)		
Brachytherapy	Prostate seed implants		
	HDR	30	Prostate, GYN
	IVBT		
	IORT		
Radiopharmaceutical therapy	[Type, Sm 153, etc]		
Other (describe)			

Other services supported or provided by physicist:

Service	Detail		Comments
Imaging physics / coordination	Ultrasound (eg, for brachytherapy guidance)		TRUS
	CT imaging quality / dose evaluations		Annual constancy evals
	MR distortion assessment (eg for SRS)		Coordinate with imaging physicist
Radiation safety	Number of radioactive materials licenses:	1	
	Serve as RSO?	No	
Computer support	Number of workstations	20	
	Type of support: networking, applications?		Applications
Administrative	Employees supervised	1	
	Responsible for physics billing policies/audits?	Yes	
	Teaching responsibilities?	No	

Clinical Physics Peer Review
Equipment and Instrumentation - RADIATION ONCOLOGY

Best RadOnc

**Date:**	23-Nov-21
**Reviewer:**	Physicist Reviewer

Clinical Equipment: Add lines if needed

Category	Manufacturer	Model	S/N	Install Date	Comments (IGRT, MLC, EPID, VMAT, robotics, #CT
Linear accelerator 1	Varian	TrueBeam	2199	10/15/15	6DOF, HDMLC
Linear accelerator 2					
CT-Simulator	Philips	BBB	1600	06/01/11	16 slice, 4DCT
Other dedicated imaging					
HDR remote afterloader	Varian	GammaMed	64A124	06/01/11	
Stereotactic radiosurgery					
Other treatment systems					
Independent X-ray system (e.g. Brainlab)					
SGRT					
Respiratory Gating	Varian	RPM			
Other					

Planning-related software systems*	Manufacturer	Model	Software version	Install Date	Options (Deformable registration, biological, auto-contouring, auto-planning etc)
System 1	Varian	Eclipse	16.1		Deformable, RapidPlan
System 2	LifeLine	RadCalc	6.3.4		Brachy, ROI
System 3					
System 4					
System 5					

	Manufacturer	Model	Software	Install date	Describe level of electronic records
Record & verify system	**Varian**	**Aria**	**16.1**		**Full EMR**
QA Database	ImageOwl	TotalQA			All electronic

Instrumentation

Category	Manufacturer	Model	Type	Last cal date	Comments
Ionization chamber 1	Std Imaging	A12	Farmer	07/11/21	
Ionization chamber 2	Std Imaging	A12	Farmer	07/15/21	Indirect
Ionization chamber 3					
Small-field detector 1					**Needed ASAP given SRS-SBRT service!**
Small-field detector 2					
Electrometer 1	PTW	UniDose		07/11/21	
Electrometer 2	Std Imaging	Max4000		06/15/20	
Well Chamber	Std Imaging	HDR1000+	Well	06/15/20	
Daily QA device	Sun Nuclear	DQA3			
IMRT QA device / system	Sun Nuclear	MapCheck2			Also portal dosimetry
3D Scanning System	PTW	BeamScan			
1D Scanning System	Sun Nuclear	1D tank			
Multidetector array					
Film dosimetry					
Calibration phantom	Gammex SNC	Solid Water			
Dynamic phantom					None - needed
Survey Meter	Ludlum	Model 3			
In-vivo dosimetry	Landauer	nanoDot	OSL		QC follows TG-191
Special-purpose phantoms					
Thermometer(s) (quantity)		Alcohol column			Two identical, intercompared
Barometer(s) (quantity)	NovaLynx	M202	Digital		Compared to NWS local station
Other phantoms					

Comments:
Describe anything about the facility that is not properly conveyed in the spaces above.
Also, *any specific aspects of the clinical physics program that the on-site physicist wishes to have the reviewer focus on* in addition to the core review components.

Clinical Physics Peer Review

Program Checklist - RADIATION ONCOLOGY

Date: 23-Nov-21
Reviewer: Physicist Reviewer

Best RadOnc

Place an "x" in the appropriate column

N/A	Yes	Conditional	No		COMMENTS
**General / Global**					
	x			Follow relevant ASTRO/ACR Practice Parameters and Technical Standards	
	x			Follow relevant AAPM TG Report & MPPG recommendations	
		x		Quality management program is appropriately comprehensive and risk-informed, and the center participates in structured Incident Learning	*Overall good QM program, but have not yet attempted to adopt TG-100 methodology for risk analysis and do not participate in Incident Learning*
	x			Physician and Physicist supervision is appropriate to scope of services	
		x		The physicist has considered "disaster recovery" options and has appropriate data backup / redundancy measures in place to enable the safe resumption of services after a significant disruption (computer networks, physical facility etc)	*Some critical files are backed up, but many are not, and backups are on a network share. Recommend a written inventory of critical files, and an off-line storage less susceptible to being compromised*
				Describe the IT network security/rights environment and the impact on the physicist's work environment (additional tasks, restricted ability to install and use specialty software etc)	*Physicist does not have local admin rights on workstations, and there is no dedicated IT liaison, which complicates installation and updates of oncology software applications.*
**State / Regulatory**					
	x			Compliant with applicable state and federal regulatory requirements.	
**Staffing Education, Credentials**					
				See Site Information tab	
	x			Appropriate reporting structure	
		x		Adequate staffing levels	*Physics staffing insufficient for new program development*
		x		Appropriate competency / credentialing procedures	*No formal process for on-boarding new staff*
**Radiation Safety / Radioactive Materials**					
	x			Radiation safety inservices are provided to all relevant staff annually	
	x			Package surveys/wipe tests are performed, and appropriate staff are trained according to	
	x			Radioactive materials are properly secured and inventoried	
**Quality Improvement**					
x				Review recommendations and action plans from any previous Peer Review. Include any unresolved issues in the current report.	
	x			Formal quality assurance & improvement program should include: chart rounds, retrospective chart audits, and morbidity & mortality reviews	
	x			Evidence that the physicist participates in QA meetings and presents documentation of QA activities	
**Major Equipment QA**					
				**External Beam Treatment Machines**	
	x			Commissioning report is available and appropriate in scope	
	x			Protocols for calibrating treatment machines follow those protocols currently published by the AAPM and adhere to state and federal guidelines.	
	x			%DD(10cm) values used for calibration factors are reasonable	
	x			An independent check of the output of each beam is performed annually (either mailed OSLD service or independent physicist check)	
		x		Monthly QA program is appropriate to the clinical utilization	*No test of gated delivery*
	x			Minimum two Radiation Therapists per machine while treating	
	x			Service and maintenance logs, and machine problem logs are consistently updated and reviewed	
				**Simulators (CT, x-Ray, PET-CT)**	
	x			Commissioning Report is available and appropriate in scope	
	x			Monthly QA program is appropriate to the clinical utilization	
**Instrumentation**					
	x			Primary instrumentation has current ADCL calibration	
	x			Secondary instruments are appropriately cross calibrated / intercompared	
	x			Barometers/thermometers are intercompared regularly	
	x			Daily QA devices are baselined against the primary instrument on a regular basis (at least annually and after any instrument repair)	
		x		Appropriate phantoms are available to test all utilized modalities (e.g. 4DCT, gated delivery, cranial SRS, SGRT, OBI/CBCT image quality and dose)	*No dynamic phantom*
**Brachytherapy**					
				**General**	
	x			Time out to verify patient identity and treatment site location	
	x			Physician and Physicist supervision during treatment	
				**HDR**	
	x			Appropriate QA program and source-strength validation methods	
	x			Appropriate safety program (area monitors, pre- and post-treatment surveys, emergency refresher training etc)	
				**LDR**	
	x			Sealed source brachytherapy data complies with standards (e.g. TG43 update (TG43U1) consensus data)	
**TPS and IMRT QA**					
				**Treatment Planning Systems (External Beam and Brachytherapy)**	
			x	Treatment planning computer model must be verified using actual beam data measured by the qualified medical physicist on the same treatment unit for which the model will be applied for patient planning	***Beam model is entirely based on Farmer chamber measurements, and no small-field validation tests were conducted after modeling. Given the recent introduction of SRS-SBRT services, this must be remedied ASAP with a suitable small-field detector and appropriate small-field validation plans.***
			x	TPS commissioning report is available and consistent with MPPG 5 recommendations	
				**IMRT**	
	x			Appropriate site-specific and fractionation-specific dose objectives	
	x			IMRT QA methods and passing criteria are consistent with TG-218 recommendations	

**SRS/SBRT**					
			x	Commissioning report is available showing relevant end-to-end process validations and clear description of overall process accuracy and any limitations	***See above regarding small-field beam modeling and validation***
		x		QA program is consistent with MPPG 9	*Need to add small-field MLC aperture test*
	x			Physician and Physicist supervision consistent with TG-101 and ACR Practice Parameter recommendations	
**Clinical program supervision**					
	x			Physics contributes to SOP development and process evalutions and improvement, including simulation and treatment delivery procedures	
		x		Physicist support for and supervision of the treatment planning process is consistent with the ACR-AAPM Technical Standard for External Beam Treatment Planning	*Physicist performs thorough and timely plan checks but is not actively engaged in developing guidance for the planning process*
	x			Physics reviews of treatment plans are consistent with the TG-275 and MPPG 11 recommendations	

Clinical Physics Peer Review

Chart Review Checklist - RADIATION ONCOLOGY

Date: 23-Nov-21

Reviewer: Physicist Reviewer

Best RadOnc

Use the following convention when scoring each chart item: N/A, Yes, Conditional, No

Select charts to be representative of the patient case mix, physicians, and treatment modalities at the center. For a single-modality, single-physician practice, 5 charts may be sufficient, whereas 10 or more charts may be appropriate for a practice with multiple physicians and complex case mix and treatment modality profile.

General for all charts	
**Treatment planning system (s)**	
Application, version	Eclipse, 16.1
Homogeneity Correction used	Yes
Second-check calculation method	RadCalc
**Record and Verify:**	
Application, version	Aria, 16.1
**IMRT QA Equipment / Procedures:**	
QA Device Used	MapCheck
Passing criteria	>95%, 2mm, 3%, true composite
**In-vivo dosimetry**	
System(s) used	OSL
Conditions for in-vivo dosimetry	Specific MD request
**Comments**	

Individual Treatment charts	Chart 1	2	3	4	5	6	7	8	9	10
Patient key *(clinic should maintain a table linking the key to the actual medical record number for potential follow-up on recommendations)*	PR1	PR2	PR3	PR4	PR5	PR6	PR7	PR8	PR9	PR10
Treatment site / Dx	Pelvis / Cervix	Prostate	H&N	Lung	Cranial mets	Esophagus	Breast - L	Prostate	Vaginal cuff	Breast - R
Type of plan: IMRT/3D/SRS/e-/HDR	IMRT/VMAT	HDR and IMRT	IMRT	SBRT	SRS	IMRT	3D/e-	IMRT	HDR	3D
MD reviewed / signed plan prior to tx	Yes	Yes	Yes	Yes	Yes	Yes	Yes	Yes	Yes	Yes
MD Rx is complete and agrees with plan**	Yes	Yes	Yes	Yes	Yes	Yes	Yes	Yes	Yes	Yes
Treatment plan documentation appropriate and clear	Yes	Yes	Yes	Yes	Yes	Yes	Yes	Yes	Yes	Yes
Setup information complete and clear, including photos**	No photos of tattoos	Yes	Yes	Yes	Yes	Yes	e- CD setup photos are	Yes	Yes	Yes
Initial physics plan review completed prior to first tx	Yes	Yes	Yes	Yes	Yes	Yes	Yes	Yes	Yes	Yes
Physics chart review & frequency are appropriate**	Yes	Yes	Yes	Yes	Yes	Yes	Yes	Yes	Yes	Yes
Therapists performed initial chart review prior to first tx	Yes	Yes	Yes	Yes	Yes	Yes	Yes	Yes	Yes	Yes
**Comments** (incl. evidence of strong safety culture)	*Clearance checks performed early in planning process*			*Beam model?*	*Beam model?*			*20 fx*	*Monotherapy*	*Hypofx no CD*

Simu	Chart 1	2	3	4	5	6	7	8	9	10
Simulation documented incl. scan range, immobilization etc	Yes	Yes	Yes	Yes	Yes	Yes	Yes	Yes	Yes	Yes
Motion management documented	N/A	N/A	N/A	Yes	N/A	Yes	Yes	N/A	N/A	N/A
**Comments**				*4D ITV*	*1mm slices*	*4D ITV*	*DIBH*			

Elect	Chart 1	2	3	4	5	6	7	8	9	10
Appropriate distance factors / cutout factors used	N/A	N/A	N/A	N/A	N/A	N/A	Yes	N/A	N/A	N/A
**Comments**							110 SSD			

IMRT	Chart 1	2	3	4	5	6	7	8	9	10
QA complete / passed prior to first tx	Yes	Yes	Yes	Conditional	Conditional	Yes	N/A	Yes	N/A	N/A
QA passing criteria documented	Yes	Yes	Yes	Yes	Yes	Yes		Yes		
MD's dose objectives and actual plan values are clearly	Yes	Yes	Yes	Yes	Yes	Yes		Yes		
**Comments**			TG-218 criteria used	**Passing criteria too loose: composite 5%/3mm**				PROFIT criteria		

IGRT/SGRT	Chart 1	2	3	4	5	6	7	8	9	10
Type used	CBCT	CBCT	CBCT	CBCT	CBCT	CBCT	MV portal	CBCT	N/A	N/A
Frequency	Daily	Daily	Daily	Daily	Daily	Daily	Daily	Daily		
Daily shifts and documentation	Yes	Yes	Yes	Yes	Yes	Yes	Yes	Yes		
**Comments**					6DOF					

SRS / SBRT	Chart 1	2	3	4	5	6	7	8	9	10
Appropriate pre treatment checks and documentation	N/A	N/A	N/A	Yes	Yes	N/A	N/A	N/A	N/A	N/A
Prior treatment assessment if applicable				N/A	Yes					
Plan evaluation metrics appropriate (eg CI, R50 etc)				Yes	Yes					
Day-of-tx machine QA (eg Winston-Lutz) performed				Yes	Yes					
Physicist and physician supervision appropriate				Yes	Yes					
**Comments**					7 cm off axis, no extra PTV margin					

Brachytherapy	Chart 1	2	3	4	5	6	7	8	9	10
Site Identified	N/A	Yes	N/A	N/A	N/A	N/A	N/A	N/A	Yes	N/A
Written Directive prior to administration		Yes							Yes	
MD approved plan		Yes							Yes	
Plan checklist / 2nd-check calculation completed		Yes							Yes	
Applicator verification		Yes							Yes	
Time Out		Yes							Yes	
Daily system QA performed		Yes							Yes	
Patient tx checklist complete for each fraction		Yes							Yes	
Survey performed and documented pre and post tx		Yes							Yes	
AU present during tx		Yes							Yes	
AMP present during tx		Yes							Yes	
Complete tx documented		Yes							Yes	
documentation		N/A							N/A	
**Comments**										

Clinical Physics Peer Review

Programmatic questions - RADIATION ONCOLOGY

Best RadOnc

**Date:**	23-Nov-21
**Reviewer:**	Physicist Reviewer

To whom does the physicist report directly?	x	Medical Director
	x	Radiation Oncology Manager
		Executive Level Administrator
		Other:
Contract Physicist?	No	
Do the dosimetrist(s) report directly to the physicist?	Yes	
Does the physicst have a formal role in the facility's budgeting and technology selection processes?	Yes	
Does the present physicist coverage provide adequate time during normal clinic hours for the physicist to consult with the treatment team regarding specific patient issues?	Yes	
Does the present physicist coverage provide adequate time during normal clinic hours for the physicist to plan and prepare for the implementation of new services?	No	
Is there adequate coverage during a physicist absence?	Contracted local physicist - inconsistent availability	
Are any new clinical physics services planned for the coming year?	Increased use of SIMT SRS and SBRT for oligomets, including new treatment sites	
Does the RadOnc practice participate in structured incident learning? Is prospective risk analysis part of the quality management program, particularly with respect to introduction of new services?	No (both)	
Does the facility provide adequate physics instrumentation, machine access and resources to support the facility's clinical service?	Need small-field detector ASAP; need dynamic phantom and more robust physics staffing	
Describe the machine service/support arrangements (hardware/software support, in-house engineers/biomed roles if applicable)	OEM service contracts, no in-house engineer	
Any other issues?		

11/26/21

Dr. Medical Director
Medical Director
Western Hospital

Dear Dr. Medical Director,

Included is a summary of the clinical physics peer review report for Western Hospital located at Street address in Hometown, AZ. The review was performed on 11/26/21 with the on-site assistance of Mr. Bremmstrahlung and was conducted in accordance with the AAPM's Medical Physics Practice Guideline (MPPG) 15 for clinical physics peer reviews.

The MPPG 15 guideline defines two categories of recommendations. 'Major recommendations' are items that, in the reviewer's opinion, do not presently meet applicable regulations or generally accepted guidelines, or scenarios that could result in harm to patients or staff. 'Minor recommendations' relate to items that have no impact on regulatory compliance, generally accepted guidelines or safety, but could enhance the physics program's productivity or level of organization.

I'm pleased to report that the review found **no** deficiencies meeting the 'Major recommendations' category in MPPG 15.

I have provided Mr. Bremsstrahlung with a list of 'Minor recommendations'. Of these, I would like to bring your attention to the following recommendations as they reflect a need for support from the institution to the physics program:
Mr. Bremmstrahlung should be provided with a set of backup test equipment for use while the primary set is being calibrated or repaired.

If you have any questions related to this report, please contact me at your convenience.

Respectfully Submitted,

[Insert Scanned Signature]

Photon, PhD
Phone number
Email address

CONFIDENTIAL

11/26/21

Mr. Bremmstrahlung
Western Hospital
City, State

Dear Incumbent physicist(s),

Thank you for the courtesy extended to me during my visit to Western Hospital on 11/26/21 for the purpose of performing a professional peer review of your clinical physics program. I found your facility to be welcoming and helpful. This appears to be a very positive environment to work in.

The review was conducted in accordance with the AAPM Medical Physics Practice Guideline 15 on clinical physics peer review. The enclosed report details the aspects of the clinical physics program that were reviewed and my findings for each component. A tour, review of quality management records and instrumentation, and conversations regarding institutional support and program needs were conducted while on site. As described in MPPG 15, the primary purpose of the peer review is to support your professional practice, and the review and resulting report were focused on that goal.

The MPPG 15 guideline defines two categories of recommendations. 'Major recommendations' are items that, in the reviewer's opinion, do not presently meet applicable regulations or generally accepted guidelines, or scenarios that could result in harm to patients or staff. 'Minor recommendations' relate to items that have no impact on regulatory compliance, generally accepted guidelines or safety, but could enhance the physics program's productivity or level of organization.

I'm pleased to report that the review found **no** deficiencies meeting the 'Major recommendations' category in MPPG 15.

The report provides a list of 'Minor recommendations' aimed at assisting you in further enhancing the clinical physics program.

I hope you find the review process, and the attached report, to be helpful to your practice. I'm available to clarify any aspect of the report.

Respectfully Submitted,

[Insert Scanned Signature]
Reviewer name, credentials
Phone number
Email address

CONFIDENTIAL

General, Operations and Facility
Your healthcare system incorporates two hospitals and six outlying imaging centers. Operating hours are appropriate for your patient population.

Staff Credentials, Coverage and Team Work
We agreed that a single Qualified Medical Physicist is appropriate staffing for this healthcare system. Access to equipment for MP testing is adequate. You are included in department-wide communications. Your ability to interact with groups outside of Imaging is excellent.

State / Regulatory
No deficiencies were found regarding your compliance with state regulations and accreditation requirements.

Radiation Safety / Radioactive Materials
No deficiencies were found with your radiation safety program. Your last radioactive materials license inspection was completed with no citations.

Quality Management
The departmental quality management program appears to be robust. You are being notified prior to any equipment purchases and participate in the equipment selection process. You are participating in safety-oriented meetings as appropriate.

Major Equipment QA
Your testing appears to be following are relevant ACR practice parameters, technical standards, and accreditation requirements. It is recommended to follow these standards for regular QC of radiographic and fluoroscopic equipment, to include radiographic repeat analysis, artifact analysis for both modalities, and manufacturer QC for both.

Instrumentation
You have the equipment you need for adequate testing, but you have no backup for when your primary equipment needs to be repaired or calibrated. It is recommended to acquire a minimal set for these occasions.

Special Procedures [If applicable]
You participate in special procedures (Y-90 therapies, primarily) as required.

Software and Networking QA
Your IT department is responsible for all software- and network-related support.

Patient-specific QA [If applicable]
You have an adequate system for responding to elevated fluoro skin exposures and pregnant patients.

Patient Chart Reviews [If applicable]
Three reports for each modality were reviewed, and are excellent. It is recommended to include pass/fail criteria for all measurements, particularly in the SPECT/CT report.

CONFIDENTIAL

New Services (Planned and Actual)
There are no new services planned in the foreseeable future.

Professionalism and Professional Development
It appears that you have a good relationship with the Imaging Department staff and with departments outside of Imaging. Your in-services are well received. You are able to regularly attend AAPM and RSNA meetings, so your continuing education and MOC are current.

Conclusions/Recommendations/Comments
Overall, you have an excellent medical physics program. If appears that you are well-supported by the department and the system, in turn, appreciates your efforts. Your testing is thorough, meeting both regulatory requirements, accreditation standards, and ACR technical standards. You are cognizant of your professional development and voluntarily participate in the ABR MOC program.

Based on the review, consistent with MPPG 15, I provide the following recommendations with the goal of supporting your professional practice and professional development:

Minor Recommendations & Comments

1. Obtain a minimal set of backup equipment
2. Include pass/fail criteria for all measurements
3. Initiate an RT QC program for radiography and fluoroscopy to include artifact analysis and repeat analysis.

Thank you again for your support of this peer review process.

Respectfully Submitted,

Reviewer name, credentials
11/26/21

CONFIDENTIAL

Clinical Physics Peer Review
Site Data

**Date of review**	26-Nov-21
**Date of previous Peer Review**	None
**Physicist Peer Reviewer**	Dr. Photon

Review previous report if applicable

**Site name**	Western Hospital
**Address**	123 Oak St
**City**	Hometown
**State**	AZ
**Zip**	12345
**Phone**	(111) 123-4567
**Incumbent physicist(s) reviewed:**	**J. Bremsstrahlung**

**Staffing** (add rows if necessary)	**Comments**	**Certified?**	**FTE**
**Physician(s)**	Contract radiology group	y	12.0
**RSO**	incumbent	y	1.0
**Physicist - Chief**	incumbent	y	1.0
**Physicist(s) - Staff**			0.0
**Physicist(s) - Consultant**			0.0
**Medical Physicist Assistant(s)**			0.0
Other staff (explain in Comments)			0.0
**Radiation Oncology staff:**			
Dosimetrist - Sr / Lead	N/A		
Dosimetrist - Staff	N/A		
Chief Therapist	N/A		
Radiation Therapist - Sr / Lead	N/A		
Radiation Therapist - Staff	N/A		
Oncology Nurse(s)	N/A		
**Imaging / Nuclear Medicine staff:**			
Mammo supervisor		y	1.0
CT supervisor		y	1.0
Radiography supervisor		y	1.0
MRI supervisor		y	1.0
Nuclear Medicine supervisor		y	1.0
Imaging Nursing supervisor		y	1.0
IR supervisor		y	1.0
Ultrasound supervisor		y	1.0
Department director		y	1.0
Department medical director		y	1.0

			**Comments**
**Clinic type:**	e.g. Free standing or Hospital, JV<br>Notes: Multiple locations, etc		
**Regional Affiliation Structure?**			2 hospitals, 6 outlying sites
**Academic affiliation?**			
**Cooperative Clinical Trails?**			

**Accreditation**	
Accrediting body used	**ACR**

Clinical Physics Peer Review
Facility Services Overview - IMAGING / NUCLEAR MEDICINE

**Date:**	26-Nov-21
**Reviewer:**	Dr. Photon

Western Hospital

Clinical services supported by physicist

Service	Sub-category	#	Adequate QC equipment available?	Comments
Imaging systems	Radiography	9	y	
	Fluoroscopy (excl. Interventional)	5	y	
	Portable radiographic	8	y	
	Mobile c-arm	8	y	
	Mammography	9	y	
	CT	7	y	
	PET-CT	1	y	no NEMA phantoms
	SPECT	0		
	SPECT-CT	4	y	
	MRI	9	y	
	Interventional (incl. cardiac cath. labs)	7	y	
	Ultrasound	19	y	
	Other			
Therapeutic / interventional		**# annually**		
	Radiopharmaceutical therapy	50		covered by NM techs
	Interventional requiring physics support	24		Y-90, present in IR room
	Radiation oncology requiring imaging physics support	1		MRI geometry
	Other (describe)			
Other (describe)				

Other services supported or provided by physicist

Service	Detail	# annually	Comments
Radiation safety	Pregnant patient safety assessments	12	
	Patient skin dose evaluations	6	
	Patient consults	12	
	Number of radioactive materials licenses:	2	
	Serve as RSO?	y	
	Serve as MRSE?	y	
Accreditation support	Number of accredited units	49	incl. US units
	Role		image upload support
Meeting attendance	Radiation Safety Committee	2	
	MR Safety Committee	2	
	CT Protocol Committee	1	
	Mammography QC Committee	1	
	Other		
Computer support	Number of workstations	21	4 mammography
	Type of support: monitor QC, networking, applications?		annual testing/calibration
Administrative	Employees supervised	0	
	Teaching responsibilities?	y	RTs

Clinical Physics Peer Review

Program Checklist - IMAGING / NUCLEAR MEDICINE

Date: 26-Nov-21
Reviewer: Dr. Photon

Western Hospital

Place an "x" in the appropriate column

N/A	Yes	Conditional	No		COMMENTS
**General / Global**					
	x			Follows relevant ACR Practice Parameters and Technical Standards	
	x			Follows relevant AAPM TG Report & MPPG recommendations	
	x			Quality management program is appropriately comprehensive and risk-informed	
	x			Physician and Physicist supervision is appropriate to scope of services (eg, interventional, radiopharmaceutical therapy etc)	
x				Has considered "disaster recovery" options and has appropriate data backup / redundancy measures in place to enable the safe resumption of services after a significant disruption (computer networks, physical facility etc)	
**State / Regulatory**					
	x			Compliant with applicable state and federal regulatory requirements.	
**Staffing Education, Credentials**					
				See Site Information tab	
	x			Appropriate reporting structure	
	x			Adequate staffing levels	
	x			Appropriate competency / credentialing procedures	
**Radiation Safety / Radioactive Materials**					
	x			Radiation safety inservices are provided to all relevant staff as required	
	x			NM audits conducted regularly	
	x			Personnel monitoring program appropriate	
	x			Shielding adequately designed and verified	
**Quality Improvement**					
x				Review recommendations and action plans from any previous Peer Review. Include any unresolved issues in the current report.	
	x			Formal quality assurance & improvement program should include: QC program audits, radiopharmaceutical treatment record audits, oversight of RT QC.	
	x			Evidence that the physicist participates in QA meetings and presents documentation of QA activities	
**Major Equipment QA**					
				**CT, SPECT, and SPECT/PET-CT systems**	
	x			Commissioning/acceptance testing report is available and appropriate in scope	
	x			Protocols for annual testing follow those protocols currently published by the AAPM and adhere to state and federal guidelines and accreditation programs.	
	x			Measured CT dose index values are reasonable [compare to CTDI DRLs?]	
	x			Task delegation and supervision are appropriate (eg, routine QC by techs or MPA)	
	x			Service and maintenance logs, and machine problem logs are consistently updated and reviewed	
				**MRI**	
	x			Acceptance testing report is available and appropriate in scope	
	x			Service and maintenance logs, and machine problem logs are consistently updated and reviewed	
	x			Protocols for annual testing follow those protocols currently published by the AAPM and adhere accreditation programs.	
	x			Routine QC performed and documented	
				**Interventional Radiology**	
	x			Patient skin doses monitored	
	x			Protocol for responding to elevated doses appropriate (incl. TJC sentinel events)	
				**X-Ray systems (radiogaphic, fluoroscopic, etc)**	
	x			Commissioning Report is available and appropriate in scope	
		x		Routine QA program is appropriate to the clinical utilization	*radiographic QC - RT flat field and repeat analysis*
				**Interpretation workstations**	
	x			Automated QC running	
	x			External photometic measurements made	
	x			GSDF conformance verified	
**Instrumentation**					
	x			Primary instrumentation has current calibration	
		x		Secondary instruments are appropriately cross calibrated / intercompared	*need backup kit*
	x			Appropriate phantoms are available to test all utilized modalities	
**MR Safety**					
	x			Available for consultations	

Clinical Physics Peer Review
Imaging Reports Checklist - IMAGING/ NUCLEAR MEDICINE

Date: 26-Nov-21
Reviewer: Dr. Photon

Western Hospital

Use the following convention when scoring each chart item:
N/A, Yes, Conditional, No

CT	Report 1	2	3
Surveyed within 13 months of prior survey	Yes	Yes	Yes
Adequate number of CT protocols reviewed	Yes	Yes	Yes
Acquisition display reviewed	Yes	Yes	Yes
Accrediting body testing requirements followed	Yes	Yes	Yes
All measurements have P/F criteria	Yes	Yes	Yes
RT QC program evaluated	Yes	Yes	Yes
CTDI compared to reference values	Yes	Yes	Yes
**Comments**			

Fluoroscopy (incl. Interventional)	Report 1	2	3
Dose rates for various body sizes measured	Yes	Yes	Yes
All dose rate modes evaluated	Yes	Yes	Yes
Local regulations followed	Yes	Yes	Yes
ACR technical standard followed	Yes	Yes	Yes
Dose rates compared to reference values	Yes	Yes	Yes
All measurements have P/F criteria	Yes	Yes	Yes
**Comments**			

Radiography	Report 1	2	3
Local regulations followed	Yes	Yes	Yes
ACR technical standard followed	Yes	Yes	Yes
Typical doses measured and compared to reference values	Yes	Yes	Yes
All measurements have P/F criteria	Yes	Yes	Yes
**Comments**			

Ultrasound	Report 1	2	3
ACR technical standard followed	Yes	Yes	Yes
All measurements have P/F criteria	Yes	Yes	Yes
**Comments**			

MRI	Report 1	2	3
Testing performed according to accrediting body requirements	Yes	Yes	Yes
Safety policies reviewed	Yes	Yes	Yes
All measurements have P/F criteria	Yes	Yes	Yes
**Comments**			

SPECT/PET-CT	Report 1	2	3
Accreditation standards followed	Yes	Yes	Yes
All measurements have P/F criteria	No	No	No
**Comments**	*Need P/F for all measurements*		

Clinical Physics Peer Review

Programmatic questions - IMAGING / NUCLEAR MEDICINE

Western Hospital

**Date:**	26-Nov-21
**Reviewer:**	Dr. Photon

To whom does the physicist report directly?		Medical Director	
	x	Radiology Manager	
		Executive Level Administrator	
		Other:	
Contract Physicist?	no		
Does the physicist have a formal role in the facility's technology assessment?	yes		
Does the present physicist coverage provide adequate time during normal clinic hours for the physicist to consult with the radiology team regarding specific equipment and/or patient issues?	yes		
Does the present physicist coverage provide adequate time during normal clinic hours for the physicist to plan and prepare for the implementation of new services?	yes		
Is there adequate physics coverage during a physicist absence?	Works with contract physicist for backup, always checks email		
Are any new clinical physics services planned for the coming year?	no		
Does the facility provide adequate physics instrumentation, machine access, and resources to support the facility's clinical service?	Need backup test kit		
What other groups does the physicist interact with? Are lines of communication open? How is in-service training provided?	Surgery, cath lab, med onc floor nurses. Training provided via on-line education modules, mostly.		
Does the imaging service utilize structured incident learning to learn from process failures? Is prospective risk analysis part of preparations for new services?	Yes, close calls reported.		
Any other issues?	None identified		

11/27/21

Dr. Medical Director
Medical Director
Eastern Hospital

Dear Dr. Medical Director,

Included is a summary of the clinical physics peer review report for Eastern Hospital located at 456 Maple Ave in Hometown, AZ. The review was performed on 11/27/21 with the on-site assistance of Mr. Brake and was conducted in accordance with the AAPM's Medical Physics Practice Guideline (MPPG) 15 for clinical physics peer reviews.

The MPPG 15 guideline defines two categories of recommendations. 'Major recommendations' are items that, in the reviewer's opinion, do not presently meet applicable regulations or generally accepted guidelines, or scenarios that could result in harm to patients or staff. 'Minor recommendations' relate to items that have no impact on regulatory compliance, generally accepted guidelines or safety, but could enhance the physics program's productivity or level of organization.

My review identified the following item meriting focused remedial attention:
Mr. Brake is seriously overworked, medical physics staff not being adequate for the volume of work required. This understaffing could lead to inadequate image quality and misdiagnosis.

I have also provided Mr. Brake with a list of 'Minor recommendations'.

If you have any questions related to this report, please contact me at your convenience.

Respectfully Submitted,

[Insert Scanned Signature]

Photon, PhD
Phone number
Email address

CONFIDENTIAL

11/27/21

Mr. Brake
Eastern Hospital
Hometown, AZ

Dear Mr. Brake,

Thank you for the courtesy extended to me during my visit to Eastern Hospital on 11/27/21 for the purpose of performing a professional peer review of your clinical physics program. The system is well-equipped and offers advanced technology and procedures for its patients.

The review was conducted in accordance with the AAPM Medical Physics Practice Guideline 15 on clinical physics peer review. The enclosed report details the aspects of the clinical physics program that were reviewed and my findings for each component. A tour, review of quality management records and instrumentation, and conversations regarding institutional support and program needs were conducted while on site. As described in MPPG 15, the primary purpose of the peer review is to support your professional practice, and the review and resulting report were focused on that goal.

The MPPG 15 guideline defines two categories of recommendations. 'Major recommendations' are items that, in the reviewer's opinion, do not presently meet applicable regulations or generally accepted guidelines, or scenarios that could result in harm to patients or staff. 'Minor recommendations' relate to items that have no impact on regulatory compliance, generally accepted guidelines or safety, but could enhance the physics program's productivity or level of organization.

My review identified one or more items meriting focused remedial attention, as described in the conclusions and recommendations section of this report.

The report also provides a list of 'Minor recommendations' aimed at assisting you in further enhancing the clinical physics program.

I hope you find the review process, and the attached report, to be helpful to your practice. I'm available to clarify any aspect of the report.

Respectfully Submitted,

[Insert Scanned Signature]
Reviewer name, credentials
Phone number
Email address

CONFIDENTIAL

General, Operations and Facility
Your healthcare system incorporates six hospitals and twelve outlying imaging centers. Several of the centers are open 24 hours, making equipment access difficult.

Staff Credentials, Coverage and Team Work
We agreed that a single Qualified Medical Physicist is not appropriate staffing for this healthcare system. There is not enough time to complete the necessary work, much less meet practice standards. Ability to interact with other departments is limited.

State / Regulatory
No deficiencies were found regarding your compliance with state regulations and accreditation requirements.

Radiation Safety / Radioactive Materials
No deficiencies were found with your radiation safety program. Your last radioactive materials license inspection was completed with several citations that have been addressed.

Quality Management
You are not always notified prior to the acquisition of new equipment. There is not adequate time to attend QA meetings.

Major Equipment QA
While your testing meets regulatory requirements, understaffing makes it difficult to meet technical standards. Due to time constraints, acceptance testing is not as complete as it should be. Oversight of RT QC is marginal, barely meeting regulatory and accreditation requirements.

Instrumentation
It appears that you are equipped with adequate instrumentation for your clinical support. Equipment is appropriately calibrated and maintained.

Special Procedures
Special procedures are left largely to the technologists, with your ability to support them limited by time constraints.

Software and Networking QA
Your IT department is responsible for all software- and network-related support.

Patient-specific QA [If applicable]
You have an adequate system for responding to elevated fluoro skin exposures and pregnant patients. Response time would be improved with better staffing.

Patient Chart Reviews [If applicable]
Three reports for each modality were reviewed, and are found to meeting regulatory, accreditation, and Joint Commission requirements, but they do not go beyond that. This could lead to inadequate image quality and missed diagnosis.

CONFIDENTIAL

New Services (Planned and Actual)
The new Y-90 program will further impact your time. Careful planning is necessary to ensure that you will be able to provide the required support.

Professionalism and Professional Development
Your limited time has curbed your ability to interact with departments outside of Imaging. In-service modules in your computer-based training system have not been updated for quite a few years, reducing their utility. While your ability to attend professional meetings is cobbled, you are managing to keep up with your continuing education and MOC.

Conclusions/Recommendations/Comments
It is clear that the understaffing issue has made it difficult, if not impossible, to provide medical physics support that is the standard of care. The recommendations below might not be able to be implemented until the staffing issue is resolved.

Based on the review, consistent with MPPG 15, I provide the following recommendations with the goal of supporting your professional practice and professional development:

Minor Recommendations & Comments

1. Improve oversight of technologist QC programs, ensuring full compliance with requirements
2. Update in-service training
3. Expand testing to comply with technical standards
4. Participate in safety meetings to provide support
5. Work with Biomed to periodically review service and preventive maintenance records
6. Expand support of interpretation workstations to at least be ensuring that the automated QC is maintaining GSDF compliance
7. Ensure that reports are forwarded to the appropriate individuals or otherwise ensure that they are kept informed of the status of their systems
8. Participate in the acquisition of new equipment, providing technical expertise to the process

Thank you again for your support of this peer review process.

Respectfully Submitted,

Reviewer name, credentials
11/26/21

CONFIDENTIAL

Clinical Physics Peer Review
Site Data

**Date of review**	27-Nov-21
**Date of previous Peer Review**	None
**Physicist Peer Reviewer**	Dr. Photon

Review previous report if applicable

**Site name**	Eastern Hospital
**Address**	456 Maple Ave
**City**	Hometown
**State**	AZ
**Zip**	67891
**Phone**	(222) 891-0123
**Incumbent physicist(s) reviewed:**	**R. Brake**

**Staffing** (add rows if necessary)	**Comments**	**Certified?**	**FTE**
**Physician(s)**	Staff radiology group	y	50.0
**RSO**	incumbent	y	1.0
**Physicist - Chief**	incumbent	y	1.0
**Physicist(s) - Staff**			0.0
**Physicist(s) - Consultant**			0.0
**Medical Physicist Assistant(s)**			0.0
Other staff (explain in Comments)			0.0
**Radiation Oncology staff:**			
Dosimetrist - Sr / Lead	N/A		
Dosimetrist - Staff	N/A		
Chief Therapist	N/A		
Radiation Therapist - Sr / Lead	N/A		
Radiation Therapist - Staff	N/A		
Oncology Nurse(s)	N/A		
**Imaging / Nuclear Medicine staff:**			
Mammo supervisor		y	1.0
CT supervisor		y	1.0
Radiography supervisor		y	1.0
MRI supervisor		y	1.0
Nuclear Medicine supervisor		y	1.0
Imaging Nursing supervisor		y	1.0
IR supervisor		y	1.0
Ultrasound supervisor		y	1.0
Department director		y	1.0
Department medical director		y	1.0

			**Comments**
**Clinic type:**	e.g. Free standing or Hospital, JV<br>Notes: Multiple locations, etc		
**Regional Affiliation Structure?**			6 hospitals, 12 outlying sites, several 24 hours
**Academic affiliation?**			yes
**Cooperative Clinical Trails?**			no

**Accreditation**	
Accrediting body used	**ACR**

Clinical Physics Peer Review

Facility Services Overview - IMAGING / NUCLEAR MEDICINE

Date:	27-Nov-21
Reviewer:	Dr. Photon

Eastern Hospital

Clinical services supported by physicist

Service	Sub-category	#	Adequate QC equipment available?	Comments
Imaging systems	Radiography	35	y	
	Fluoroscopy (excl. Interventional)	20	y	
	Portable radiographic	16	y	
	Mobile c-arm	18	y	
	Mammography	21	y	
	CT	18	y	
	PET-CT	4	y	no NEMA phantoms
	SPECT	2	y	
	SPECT-CT	7	y	
	MRI	18	y	
	Interventional (incl. cardiac cath. labs)	20	y	
	Ultrasound	43	y	
	Other			
Therapeutic / interventional		**# annually**		
	Radiopharmaceutical therapy	50		covered by NM techs
	Interventional requiring physics support	75		Y-90, present in IR room
	Radiation oncology requiring imaging physics support	4		MRI geometry
	Other (describe)			
Other (describe)				

Other services supported or provided by physicist

Service	Detail	# annually	Comments
Radiation safety	Pregnant patient safety assessments	20	
	Patient skin dose evaluations	12	
	Patient consults	24	
	Number of radioactive materials licenses:	6	
	Serve as RSO?	y	
	Serve as MRSE?	y	
Accreditation support	Number of accredited units	113	incl. US units
	Role		image upload support
Meeting attendance	Radiation Safety Committee	6	
	MR Safety Committee	6	
	CT Protocol Committee	3	
	Mammography QC Committee	0	
	Other		
Computer support	Number of workstations	48	4 mammography
	Type of support: monitor QC, networking, applications?		annual testing/calibration
Administrative	Employees supervised	0	
	Teaching responsibilities?	y	RTs

Clinical Physics Peer Review

Program Checklist - IMAGING / NUCLEAR MEDICINE

Date: 27-Nov-21
Reviewer: Dr. Photon

Eastern Hospital

Place an "x" in the appropriate column

N/A	Yes	Conditional	No	Item	COMMENTS
**General / Global**					
		x		Follows relevant ACR Practice Parameters and Technical Standards	
		x		Follows relevant AAPM TG Report & MPPG recommendations	
	x			Quality management program is appropriately comprehensive and risk-informed	
	x			Physician and Physicist supervision is appropriate to scope of services (eg, interventional, radiopharmaceutical therapy etc)	
x				Has considered "disaster recovery" options and has appropriate data backup / redundancy measures in place to enable the safe resumption of services after a significant disruption (computer networks, physical facility etc)	
**State / Regulatory**					
	x			Compliant with applicable state and federal regulatory requirements.	
**Staffing Education, Credentials**					
				See Site Information tab	
		x		Appropriate reporting structure	
			x	Adequate staffing levels	
	x			Appropriate competency / credentialing procedures	
**Radiation Safety / Radioactive Materials**					
	x			Radiation safety inservices are provided to all relevant staff as required	
	x			NM audits conducted regularly	
	x			Personnel monitoring program appropriate	
	x			Shielding adequately designed and verified	
**Quality Improvement**					
x				Review recommendations and action plans from any previous Peer Review. Include any unresolved issues in the current report.	
			x	Formal quality assurance & improvement program should include: QC program audits, radiopharmaceutical treatment record audits, oversight of RT QC.	
		x		Evidence that the physicist participates in QA meetings and presents documentation of QA activities	
**Major Equipment QA**					
				**CT, SPECT, and SPECT/PET-CT systems**	
		x		Commissioning/acceptance testing report is available and appropriate in scope	
		x		Protocols for annual testing follow those protocols currently published by the AAPM and adhere to state and federal guidelines and accreditation programs.	
	x			Measured CT dose index values are reasonable [compare to CTDI DRLs?]	
	x			Task delegation and supervision are appropriate (eg, routine QC by techs or MPA)	
			x	Service and maintenance logs, and machine problem logs are consistently updated and reviewed	
				**MRI**	
	x			Acceptance testing report is available and appropriate in scope	
			x	Service and maintenance logs, and machine problem logs are consistently updated and reviewed	
	x			Protocols for annual testing follow those protocols currently published by the AAPM and adhere accreditation programs.	
	x			Routine QC performed and documented	
				**Interventional Radiology**	
	x			Patient skin doses monitored	
	x			Protocol for responding to elevated doses appropriate (incl. TJC sentinel events)	
				**X-Ray systems (radiogaphic, fluoroscopic, etc)**	
		x		Commissioning Report is available and appropriate in scope	
		x		Routine QA program is appropriate to the clinical utilization	
				**Interpretation workstations**	
	x			Automated QC running	
			x	External photometic measurements made	
			x	GSDF conformance verified	
**Instrumentation**					
	x			Primary instrumentation has current calibration	
	x			Secondary instruments are appropriately cross calibrated / intercompared	
	x			Appropriate phantoms are available to test all utilized modalities	
**MR Safety**					
		x		Available for consultations	

Clinical Physics Peer Review

Imaging Reports Checklist - IMAGING/ NUCLEAR MEDICINE

Date: 27-Nov-21
Reviewer: Dr. Photon

Eastern Hospital

Use the following convention when scoring each chart item:
N/A, Yes, Conditional, No

CT	Report 1	2	3
Surveyed within 13 months of prior survey	Yes	Yes	Yes
Adequate number of CT protocols reviewed	No	No	No
Acquisition display reviewed	Yes	Yes	Yes
Accrediting body testing requirements followed	Yes	Yes	Yes
All measurements have P/F criteria	No	No	No
RT QC program evaluated	Yes	Yes	Yes
CTDI compared to reference values	No	No	No
**Comments**			

Fluoroscopy (incl. Interventional)	Report 1	2	3
Dose rates for various body sizes measured	Yes	Yes	Yes
All dose rate modes evaluated	No	No	No
Local regulations followed	Yes	Yes	Yes
ACR technical standard followed	No	No	No
Dose rates compared to reference values	Yes	Yes	Yes
All measurements have P/F criteria	No	No	No
**Comments**			

Radiography	Report 1	2	3
Local regulations followed	Yes	Yes	Yes
ACR technical standard followed	No	No	No
Typical doses measured and compared to reference values	No	No	No
All measurements have P/F criteria	No	No	No
**Comments**			

Ultrasound	Report 1	2	3
ACR technical standard followed	No	No	No
All measurements have P/F criteria	No	No	No
**Comments**			

MRI	Report 1	2	3
Testing performed according to accrediting body requirements	Yes	Yes	Yes
Safety policies reviewed	Yes	Yes	Yes
All measurements have P/F criteria	No	No	No
**Comments**			

SPECT/PET-CT	Report 1	2	3
Accreditation standards followed	Yes	Yes	Yes
All measurements have P/F criteria	No	No	No
**Comments**	*Need P/F for all measurements. Meet minimum*		

Clinical Physics Peer Review

Programmatic questions - IMAGING / NUCLEAR MEDICINE

Eastern Hospital

**Date:**	27-Nov-21
**Reviewer:**	Dr. Photon

To whom does the physicist report directly?		Medical Director
	x	Radiology Manager
		Executive Level Administrator
		Other:
Contract Physicist?		no
Does the physicist have a formal role in the facility's technology assessment?		no
Does the present physicist coverage provide adequate time during normal clinic hours for the physicist to consult with the radiology team regarding specific equipment and/or patient issues?		no
Does the present physicist coverage provide adequate time during normal clinic hours for the physicist to plan and prepare for the implementation of new services?		no
Is there adequate physics coverage during a physicist absence?		Works with contract physcist for backup, always checks email
Are any new clinical physics services planned for the coming year?		Yes, Y-90 program to start
Does the facility provide adequate physics instrumentation, machine access, and resources to support the facility's clinical service?		No, access to some outside clinics is limited
What other groups does the physicist interact with? Are lines of communication open? How is in-service training provided?		Surgery, cath lab, med onc floor nurses. Training provided via on-line education modules, mostly.
Does the imaging service utilize structured incident learning to learn from process failures? Is prospective risk analysis part of preparations for new services?		Yes, close calls reported.
Any other issues?		Seriously understaffed. MP has no time for going beyond minimal regulatory and accreditation requirements.
