# Supplementary material for: AAPM Medical physics practice guideline 15.A: Peer review in clinical physics
Source: J Appl Clin Med Phys. 2023 Sep 14;24(10):e14151. doi: 10.1002/acm2.14151 (PMC10562014; doi:10.1002/acm2.14151)
Supplement: Supplementary file 2 — Peer Review Report Template [file ACM2-24-e14151-s003.docx]

## *[Template Cover Letter to Medical Director – delete this note]*

Date of report

## Dr. Medical Director

Medical Director

Facility name

## Dear Dr. Medical Director,

## Included is a summary of the clinical physics peer review report for Facility name located at Street address in City, State. The review was performed on Review date with the on-site assistance of Incumbent physicist(s) and was conducted in accordance with the AAPM’s Medical Physics Practice Guideline (MPPG) 15 for clinical physics peer reviews.

## The MPPG 15 guideline defines two categories of recommendations. ‘Major recommendations’ are items that, in the reviewer’s opinion, do not presently meet applicable regulations or generally accepted guidelines, or scenarios that could result in harm to patients or staff. ‘Minor recommendations’ relate to items that have no impact on regulatory compliance, generally accepted guidelines or safety, but could enhance the physics program’s productivity or level of organization.

## *[Use one of the following statements and delete the one which is not applicable, as well as this note]*

## I’m pleased to report that the review found no deficiencies meeting the ‘Major recommendations’ category in MPPG 15. My review identified the following items meriting focused remedial attention:

## I have provided Incumbent physicist(s) with a list of ‘Minor recommendations’. Of these, I would like to bring your attention to the following recommendations as they reflect a need for support from the institution to the physics program:

## *Outline the recommendations requiring institution support here, either in narrative or list form*

## If you have any questions related to this report, please contact me at your convenience.

## Respectfully Submitted,

[Insert Scanned Signature]

## Reviewer name, credentials

Phone number

Email address

***[Template Cover Letter to Incumbent Physicist(s) – delete this note]***

## Date of report

Incumbent physicist(s)

Facility name

City, State

Dear Incumbent physicist(s),

Thank you for the courtesy extended to me during my visit to Facility name on Review date for the purpose of performing a professional peer review of your clinical physics program. Write a single sentence or two, something positive about the site: e.g. “The documentation of your program is excellent and made the review process quite enjoyable.”

The review was conducted in accordance with the AAPM Medical Physics Practice Guideline 15 on clinical physics peer review. The enclosed report details the aspects of the clinical physics program that were reviewed and my findings for each component. A tour, review of quality management records and instrumentation, and conversations regarding institutional support and program needs were conducted while on site. As described in MPPG 15, the primary purpose of the peer review is to support your professional practice, and the review and resulting report were focused on that goal.

## The MPPG 15 guideline defines two categories of recommendations. ‘Major recommendations’ are items that, in the reviewer’s opinion, do not presently meet applicable regulations or generally accepted guidelines, or scenarios that could result in harm to patients or staff. ‘Minor recommendations’ relate to items that have no impact on regulatory compliance, generally accepted guidelines or safety, but could enhance the physics program’s productivity or level of organization.

## *[Use one of the following statements and delete the one which is not applicable, as well as this note]*

## I’m pleased to report that the review found no deficiencies meeting the ‘Major recommendations’ category in MPPG 15. My review identified one or more items meriting focused remedial attention, as described in the conclusions and recommendations section of this report.

The report provides a list of ‘Minor recommendations’ aimed at assisting you in further enhancing the clinical physics program.

I hope you find the review process, and the attached report, to be helpful to your practice. I’m available to clarify any aspect of the report.

## Respectfully Submitted,

[Insert Scanned Signature]

## Reviewer name, credentials

Phone number

Email address

**General, Operations and Facility**

Provide a high-level summary of the facility and clinical scope of services, such as the number of patients served, key equipment, overall staffing levels and structure etc.

**Staff Credentials, Coverage and Team Work**

Describe the physician and physics staffing model and credentials and RSO arrangement, overall staffing levels of allied health staff, and high-level observations regarding clinical workflow, staff meetings and communication etc.

**State / Regulatory**

See program checklist. If no deficiencies, specifically state that no deficiencies were found.

**Radiation Safety / Radioactive Materials**

See program checklist to determine if anything requires comment.

**Quality Management**

See program checklist – reflect both positive and negative observations

**Major Equipment QA**

See program checklist – reflect both positives and opportunities for program enhancement

**Instrumentation**

See program checklist, and consider whether to recommend additional instrumentation

**Special Procedures** [If applicable]

Eg, SRS-SBRT or HDR, or interventional procedures or radiopharmaceutical therapy

**Software and Networking QA**

Comment on validation and routine QA of critical software systems, process for system upgrades, data security etc

**Patient-specific QA** [If applicable]

Eg, IMRT QA for RadOnc practices

**Patient Chart Reviews** [If applicable]

Summarize number and categories of charts reviewed. Include both positives and opportunities for improvement.

**New Services (Planned and Actual)**

Based on interviews, comment on new services and the impact on clinical physics support – both staffing level and instrumentation - and required training etc

**Professionalism and Professional Development**

Comment on the physicists’ “soft skills”, leadership within the clinical team environment, contribution to the institution’s success, and commitment to professional development.

**Conclusions/Recommendations/Comments**

# Start with a high-level impression of the program and the physicists’ professionalism and skill set, stating the positives first.

Based on the review, consistent with MPPG 15, I provide the following recommendations with the goal of supporting your professional practice and professional development:

# Major Recommendations *[Expected to be rare; in many cases, delete this section]*

2. etc

**Minor Recommendations & Comments**

2. etc

Thank you again for your support of this peer review process.

## Respectfully Submitted,

## Reviewer name, credentials

Date of report
